# Epistemic agency in self-tracking: a conceptual literature review of quantified self as citizen science for health

**DOI:** 10.3389/fpubh.2026.1736850

**Published:** 2026-08-18

**Authors:** Sara Riggare, Thomas Blomseth Christiansen, Jakob Eg Larsen, Steven Jonas, Gary Isaac Wolf, Martijn de Groot

**Affiliations:** 1 Participatory eHealth and Health Data, Department for Women's and Children's Health, Uppsala University, Uppsala, Sweden; 2 Konsulent Blomseth, Hjørring, Denmark; 3 Department of Applied Mathematics and Computer Science, Technical University of Denmark, Kongens Lyngby, Denmark; 4 Article 27 Foundation, Berkeley, CA, United States; 5 Health Innovation Labs, Radboud University Medical Centre, Nijmegen, Netherlands

**Keywords:** citizen science, citizen science for health, epistemic agency, personal science, quantified self, self-tracking

## Abstract

**Introduction:**

Citizen science for health invites non-professionals into research, but the degree to which individuals control the knowledge-making process varies widely. Personal science—the self-directed, inquiry-driven practice associated with the Quantified Self (QS) community—represents a high-agency form of self-tracking in which the individual is both subject and scientist. Drawing on work on epistemic injustice, we use “epistemic agency” to describe whether a person exercises genuine control over inquiry rather than merely supplying data.

**Aim:**

To assess the utility of an agency-based framework for characterizing the epistemic dimension of self-tracking, and to examine how personal science has been represented in the scientific literature.

**Methods:**

Following Jaakkola's theory-synthesis approach, we developed a four-phase analytical framework (questioning, observing, reasoning, discovering) with three levels of individual agency (low agency, mixed agency, high agency). A search of Web of Science and PubMed (April–May 2025) for “quantified self” yielded 721 records; 241 were retained for analysis, of which 46 used self-tracking as the data-collection method and were classified by agency level through independent dual-author review.

**Results:**

The 46 articles spanned the full agency spectrum: 17 low, 10 mixed, and 19 high. Low-agency studies treated individuals as data sources, whereas high-agency studies displayed the complete self-directed cycle, often with the self-tracker as co-author. Data-sharing dominated low-agency accounts, while sharing of methods and experiences characterized higher-agency work.

**Conclusion:**

The framework can meaningfully distinguish merely measurement from self-directed knowledge production, turning on *who owns the question*. Personal science can be seen as a high-agency dimension of citizen science for health.

## 1 Introduction

Citizen participation in science and public health has a long tradition and reflects a commitment to democratizing knowledge and recognizing diverse forms of expertise. Citizen science, meaning the active engagement of non-professionals in research, has long served to expand data collection and empower communities in fields such as environmental monitoring, where amateur participation in biodiversity surveys is common. However, in health and healthcare, this field of research is relatively underdeveloped compared to biomedical science by professional researchers and clinicians. The inclusion of patients and the public in health research has been formalized through a range of established frameworks, including participatory health research (PHR) (1), patient and public involvement and engagement (PPI or PPIE) (2, 3), patient-oriented research (POR) (4), community-based participatory research (CBPR) (5), patient engagement (6), and patient innovation (7). One approach that is the specific attention of this article is the Quantified Self (QS).

The QS movement emerged in 2008 as an informal community-driven initiative exploring “self-knowledge through numbers” (8). Rooted in the empirical tradition of self-experimentation (9–11), QS practitioners use digital and non-digital tools for structured observation to investigate and reflect on personally meaningful questions about health and wellbeing. From studying the QS community, Heyen proposed using the term personal science for the production of “verified and practical self-knowledge” (12, 13). Meanwhile, Wolf and de Groot (8) presented a framework for personal science, based on practices in the QS community. This framework defined five cyclical activities: Questioning, Designing, Observing, Reasoning, and Discovering, through which individuals use empirical methods to explore personally consequential questions (8). Each phase centers on activities with high epistemic agency: the person chooses what to ask, what and how to collect and analyze data, and how to consolidate and share findings in ways applicable to their own daily life.

Across established participation frameworks, the degree to which citizens and patients exercise control over the research process varies considerably (14, 15). Den Broeder et al. (16) proposed a typology of citizen science, from contributory data collection (low agency) to what, following Haklay, is termed “extreme citizen science” (17), with citizens leading all stages of inquiry. A complementary typology of self-tracking activities was proposed by Heyen (13), who distinguished four levels of engagement that map onto the citizen science spectrum. These levels are: *no concrete goals, monitoring & optimization, research*, and *research & development* and Heyen links all but level two to citizen science and personal science (13).

Personal science, as a conceptual label for the practices seen in the QS community, shares features with citizen science. Non-professionals are engaging in structured inquiry and production of knowledge (13). Participants in citizen science and personal science reject the idea that valid knowledge can only be produced by credentialed experts, and hold that knowledge grounded in lived experience is not merely anecdotal noise but has genuine epistemic value (8, 12, 16, 18–20). However, in citizen science for health, researchers or public health bodies typically invite non-professionals into a pre-existing research structure, defining the questions and retaining analytical authority (16, 18, 19). Even in highly participatory models of citizen science, power-sharing may be deliberately managed by institutional actors (16). Personal science, by contrast, is self-directed: the individual generates the question, designs the inquiry, and determines what counts as a meaningful answer (8, 12).

Based on Miranda Fricker's work on epistemic injustice, we propose to use the label “epistemic agency” to describe not merely whether a person participates in research, but whether they exercise genuine control over the knowledge-making process itself (18, 19, 21). The concept of epistemic agency has been developed chiefly in philosophy and education, where it names a person's standing as a producer and evaluator of knowledge rather than a passive recipient of it. As the philosopher Catherine Elgin defined it: “Epistemic agents should think of themselves as, and act as, legislating members of a realm of epistemic ends: they make the rules, devise the methods, and set the standards that bind them” (22). A related body of work on epistemic injustice in a healthcare context describes what is lost when that standing goes unrecognized: people can be wronged specifically in their capacity as knowers, including damage to their own ability to make sense of their experience (23). Here, we bring these strands together and apply them specifically to citizen science for health. By epistemic agency we mean the capacity of an individual to act as a producer of knowledge rather than only a source of data—to pose questions, gather and interpret evidence, reach conclusions, and be recognized by others as a credible knower.

We believe that clarifying the concept of epistemic agency in self-tracking practices would benefit both personal science and citizen science for health. Without conceptual clarity it becomes difficult to support individuals as legitimate producers of health knowledge (18, 24). Therefore, we developed a framework, built on the personal science cycle, for assessing epistemic agency in self-tracking research by distinguishing three levels of individual control across four phases of empirical inquiry. The aim of this study was to assess the utility of this framework for characterizing the epistemic dimension of self-tracking, by using it to classify studies across a body of literature. In doing so, we also examine how personal science—the participatory, inquiry-driven, high-agency form of self-tracking—has been represented in that literature.

## 2 Material and methods

### 2.1 Research design

Following Jaakkola's framework for conceptual papers, this study is designed as a theory synthesis, which seeks to achieve conceptual integration by analysis through a particular theoretical lens (25). Here, the scientific literature constitutes the domain theory—the substantive body of knowledge under examination—while the conceptual framework for personal science (8) serves as the method theory, providing the analytical lens through which that literature is organized and evaluated. This design is appropriate because the relevant literature is fragmented across disciplinary perspectives that employ differing framings of the same phenomenon, and theory synthesis is particularly suited to structuring such fragmentation by identifying commonalities and distinctions (25).

Our active involvement in the field means that we bring detailed experience and knowledge as well as potential biases to this study. As insiders to the QS community, we have direct, longitudinal knowledge of its practices, values, and self-understanding that would be difficult to obtain from the literature alone. This positioned us to apply the conceptual framework for personal science with the interpretive depth that comes from having contributed to its development. At the same time, our investment in the QS community and in personal science as a concept means that we approached this review with prior commitments about what the phenomenon is and why it matters. We acknowledge in the limitations section ways in which our positionality may have shaped our interpretations.

### 2.2 Analytical framework

To serve as the method theory (25) for this review, we developed an analytical framework for epistemic agency in self-tracking research based on the conceptual framework for personal science (8). We used an adapted four-phase personal science framework instead of the original five phases. This modification is based on our experiences from conducting, teaching, and evaluating personal science projects. We have found that activities during the original second phase “Designing” are tightly interwoven with the subsequent phase “Observing.” For example, tool choice and measurement definitions typically evolve during first data collection rather than in advance. We therefore merged the original second with the third phase, leading to a framework with four instead of five phases. This four-phase scheme better matches personal science workflows. Here, we labeled the phases: Questioning: developing overall goals and questions, Observing: defining measurable and relevant phenomena as well as collecting data, Reasoning: analysis and visualization of data, and Discovering: articulating what was learned, which often leads to more questions. These phases are often described as a cycle, starting with Questioning. In personal science, all phases are undertaken by the person doing the self-tracking.

**Figure 1:**
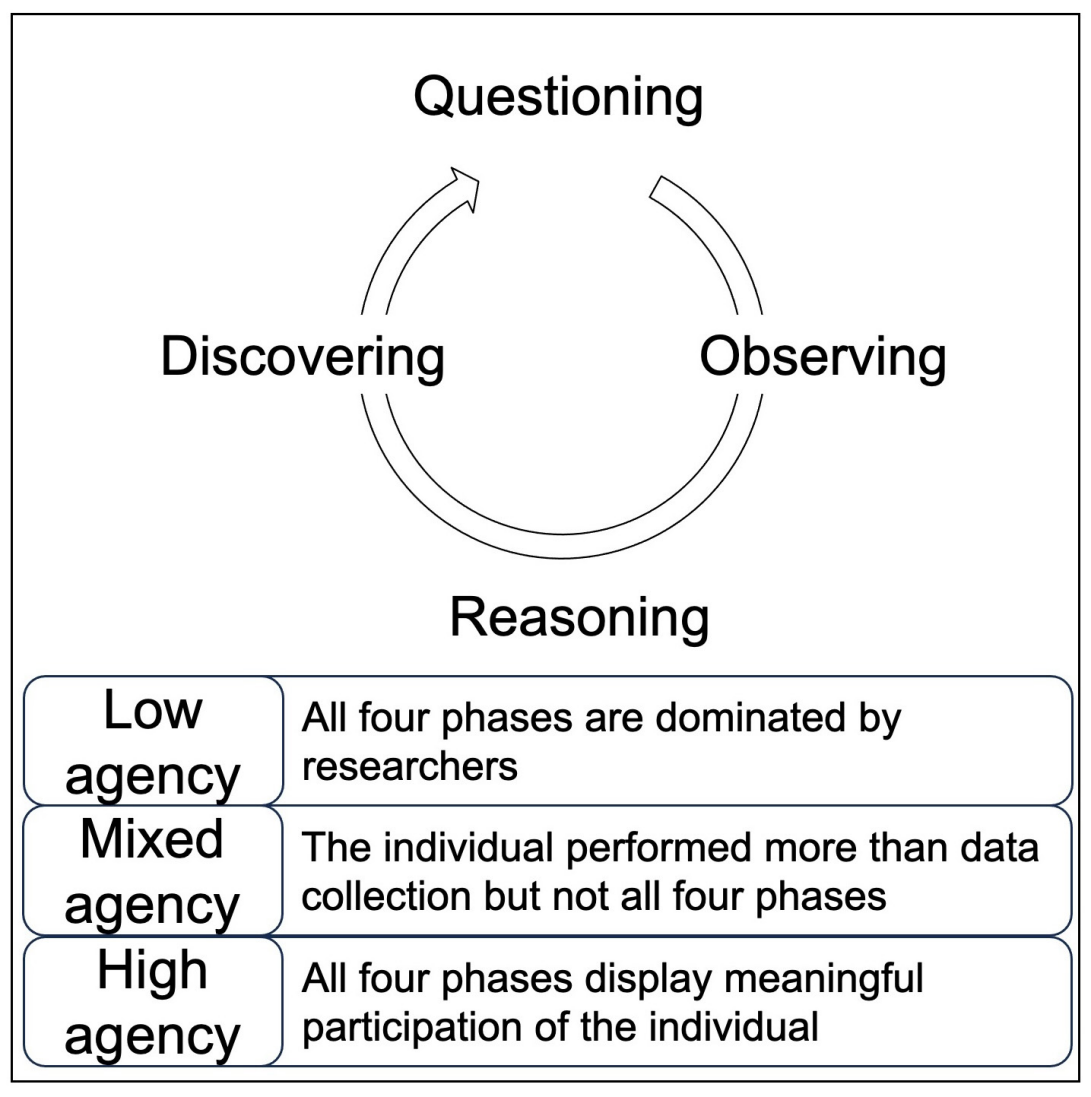
Analytical framework, based on the Personal Science Cycle [adapted from (8)].

Our analytical framework was designed with three levels of agency. In low-agency articles, only the data-collection component of Observing can be attributed to the individual; all other phases are performed by researchers. Mixed-agency articles are those in which the individual performed more than data collection alone, for instance, by engaging in self-defined monitoring goals or reflective interpretation of data, but do not demonstrate all four phases. In high-agency articles, all four phases can be identified as performed by or with meaningful participation of the individual doing the self-tracking. See Figure 1 for a representation of the analytical framework.

### 2.3 Literature search and screening

We conducted a review of “quantified self” in the scientific literature in health, medicine, and biomedical science. “quantified self” was chosen as the search term rather than alternatives such as “personal science” or “self-tracking” for reasons that are both conceptual and practical. Conceptually, the QS community is the empirical site from which personal science was derived: both the conceptual framework for personal science (8) and Heyen's characterization of self-related knowledge production (12) were built directly from study of QS practices, making it the primary real-world instantiation of personal science. Practically, “personal science” remains too sparsely used in the literature to return a representative corpus, while “self-tracking” is too broad. “quantified self” occupies a more precise conceptual position while being established enough in the literature to yield a meaningful body of records.

A search conducted in Web of Science and PubMed in April–May 2025 using the search term “quantified self” in the fields Author Keyword, Title, or Abstract (Web of Science) and all fields (PubMed) yielded 721 unique records. The review of identified records was carried out in stages. We were looking for peer-reviewed original research on the use of self-tracking (in both conventional research and in personal science). First, one author (SR) excluded records that were; not in English, lacking an abstract, or the wrong article type. Second, abstracts of the remaining 618 records were reviewed by all authors. Each abstract was read and categorized by two authors independently. Abstracts were excluded if the term “quantified self” was used in a different context (for example: “we quantified self-reported symptoms…”), if the main purpose was to describe development of technology intended for QS activities (rather than using those tools), or if the article type was out of scope (review article, position paper, description of workshop etc). These criteria were designed to capture peer-reviewed literature that engages with self-tracking as an empirical practice—the domain in which epistemic agency can be meaningfully assessed—while excluding studies where “quantified self” was used incidentally or where the primary purpose was tool development rather than research involving individuals. The inclusion of both conventional research studies and personal science publications reflects the study's aim of mapping the full spectrum of epistemic agency representations, from researcher-directed data collection to fully self-directed inquiry. Excluding review articles, position papers, and workshop descriptions ensured that the corpus consisted of primary research from which agency attributions could be directly assessed rather than synthesized from secondary sources.

**Figure 2:**
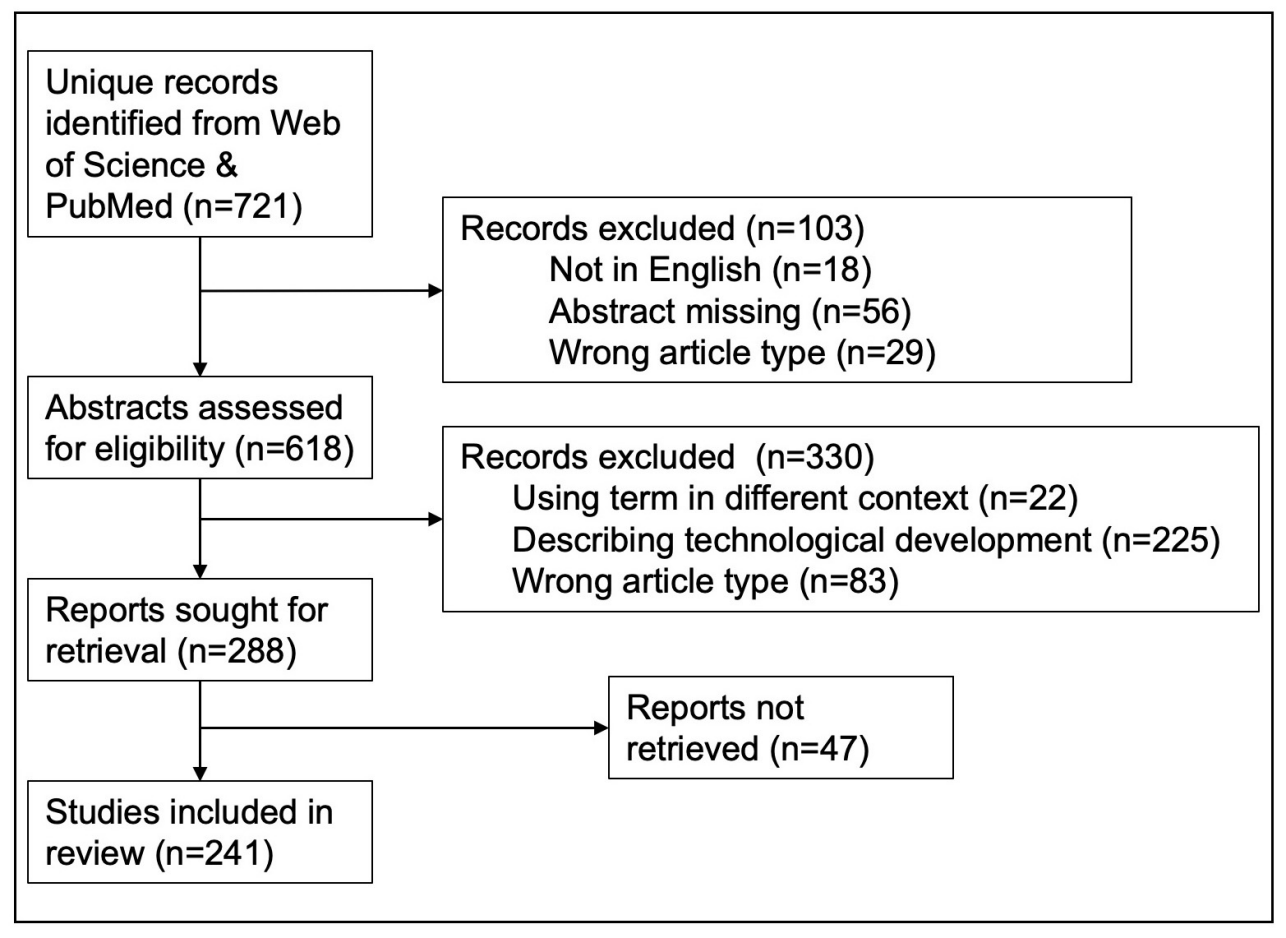
Flow diagram showing the screening process.

In 13% of cases (*n* = 80), the two reviewers did not agree, and these conflicts were resolved through discussion among all authors. Thereafter author SR retrieved all eligible records, yielding a total number of 241 full papers for final analysis. See also the flow diagram of the screening process in Figure 2.

### 2.4 Categorization and analysis

Full papers (*n* = 241) were read, categorized, and analyzed in a stepwise approach led by author SR in collaboration with author MdG with input from all authors. The first categorization separated articles containing no empirical data from individuals, including theoretical papers, critical reflections, method development, philosophical discussions, opinion pieces, commentaries, and discourse analyses, from those that did. This distinction was necessary because only articles containing data from individuals could be meaningfully assessed for levels of epistemic agency. Among the articles containing empirical data from individuals, a second categorization distinguished between studies where data were collected using methods external to self-tracking, such as surveys, interviews, or researcher-administered observations, and studies where data were generated by individuals through self-tracking. Only articles in which self-tracking was the data collection method (*n* = 46) were analyzed using the four-phase framework to assess who performed each phase of inquiry.

Finally, the articles where data were collected using self-tracking were subjected to a detailed analysis based on the analytical framework described earlier. We examined which phases of empirical inquiry were attributed to the individual vs. the researcher using our analytical framework. The 46 articles were assessed by author SR and at least one other author by asking, for each of the four phases, whether it was performed by the individual doing the self-tracking or by the researcher. Observing was present in all articles by definition, since data collection by self-tracking was the inclusion criterion for this analytical step. For Questioning, articles were assessed for whether the research question or tracking goal was formulated by the individual, emerging from their own curiosity, symptoms, or personal priorities, or whether it was defined by the investigators. For Reasoning, articles were assessed for whether the individual performed or meaningfully participated in the analysis and interpretation of their own data, or whether this was conducted solely by researchers. For Discovering, articles were assessed for whether the individual articulated what was learned and determining how findings were applied to their own life, including whether sharing of results was self-initiated.

In practice, high-agency articles were often distinguished by the fact that the self-tracker was also a co-author of the scientific publication, which served as an indicator that the Discovering phase, including the articulation and dissemination of findings, was self-directed.

Where an article contained elements that could be read as belonging to more than one level, the lower level was assigned unless there was clear textual evidence that the individual had exercised agency in the disputed phase. This conservative approach was adopted to avoid overstating the degree of epistemic agency present in the literature.

In 17% of cases (*n* = 8), reviewers did not agree, and cases where the analysis differed were resolved by consulting all authors.

## 3 Results

**Figure 3:**
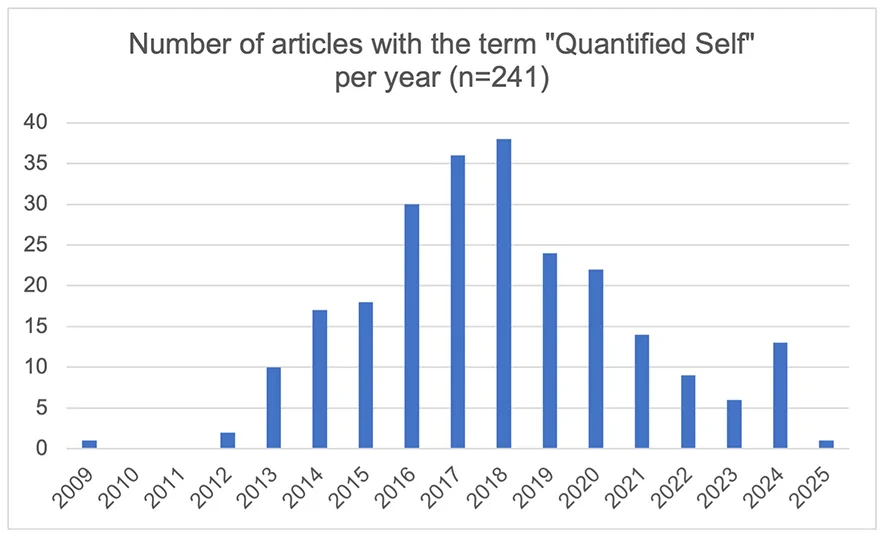
Histogram of number of retrieved full papers with the term “quantified self” per publication year.

**Figure 4:**
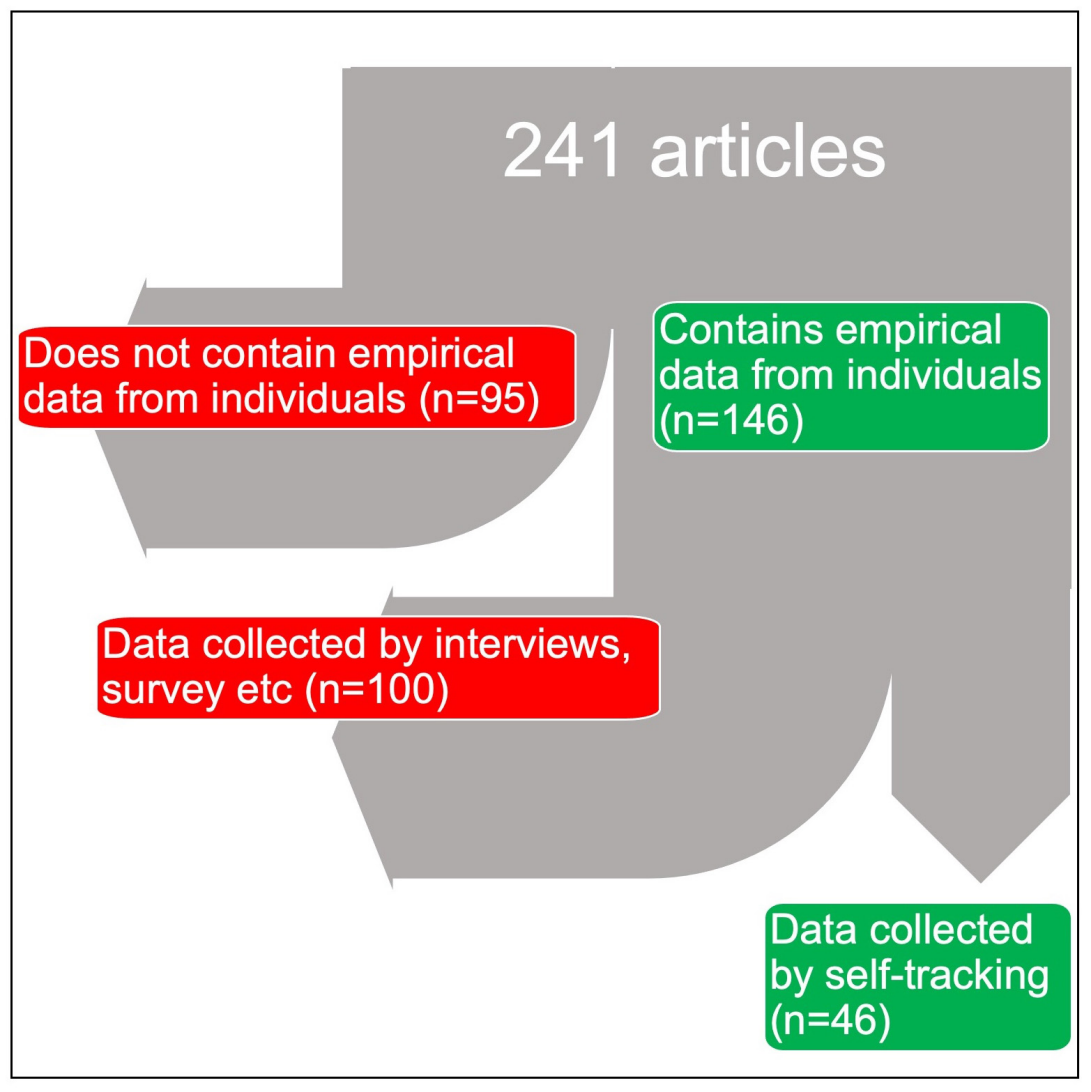
Categorization of the included articles with the term “quantified self”.

In total 241 full papers with the term quantified self were retrieved, see Supplementary Table 1 for a list of the papers and Figure 3 for a graph of the number of papers per publication year. There was a peak in 2018 with 38 published articles. In recent years there seems to be an uptick with 2024 (13 articles published) almost reaching the numbers in 2021 (14 articles). A visual representation of the categorization of the included 241 papers is depicted in Figure 4.

### 3.1 Levels of agency

The articles where data were collected by means of self-tracking (*n* = 46) were analyzed and subdivided into different levels of agency using the framework for personal science, as described under Methods. The articles were found to encompass a wide spectrum of agency on the part of the individual doing the tracking, ranging from passive participation in externally defined research to fully self-directed inquiry. All papers included the collecting data part of Observing, but inclusion of the other phases (Questioning, Reasoning, and Discovering), varied greatly.

The articles further differ in analytical focus: some articles focus on the individuals doing the self-tracking, which we refer to as “articles describing self-trackers (the people).” Other articles focus on the activity of self-tracking, and we refer to these as “articles describing self-tracking (the activity). Distinguishing between these two perspectives is important because it clarifies whether articles are about the community or about the tracking process itself.

**Figure 5:**
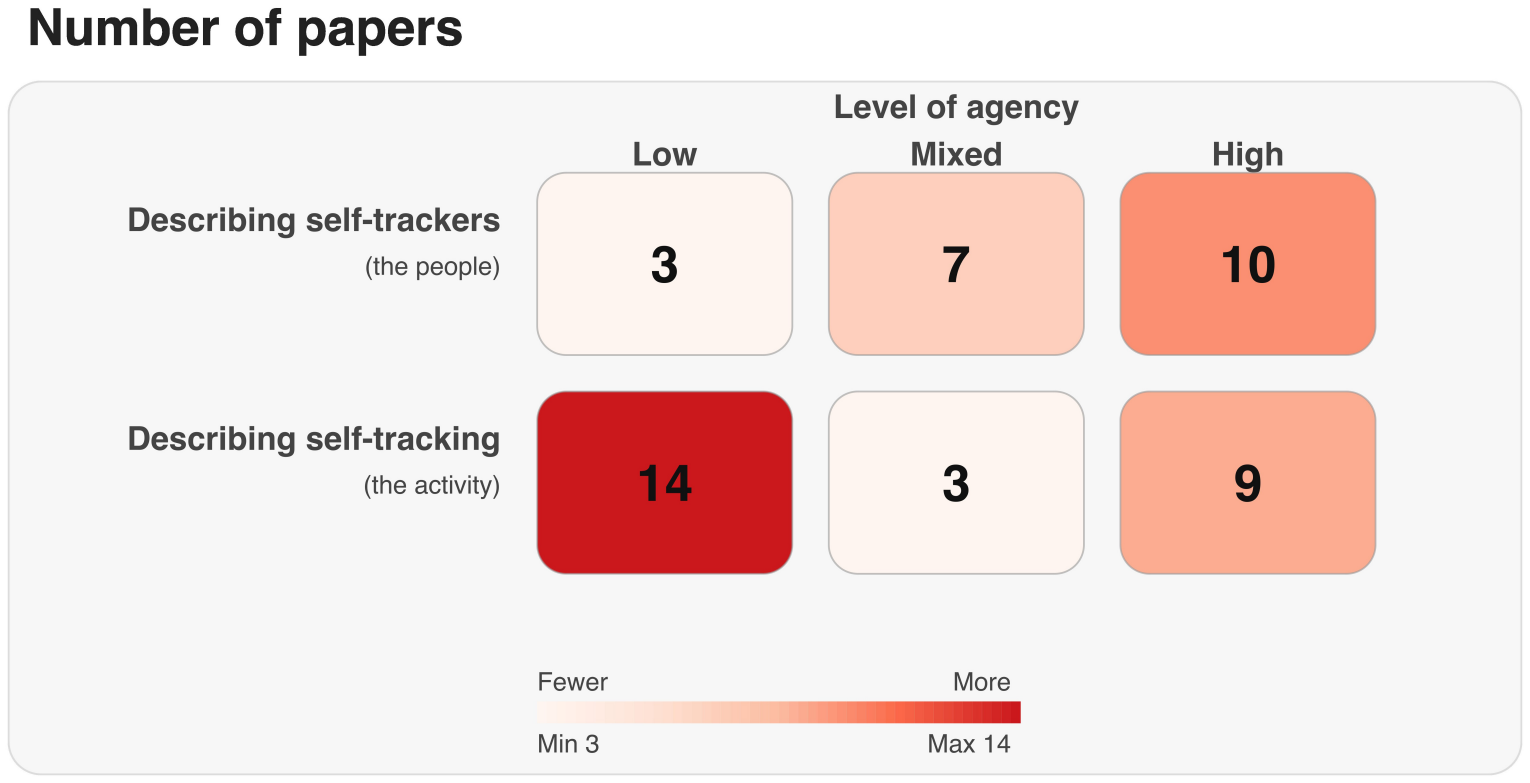
Heatmap of number of articles per level of agency (low/mixed/high) for articles describing self-trackers (the people) and self-tracking (the activity) respectively.

See Figure 5 for number of articles per level of agency for articles describing self-trackers and self-tracking respectively, illustrated in a heatmap and Supplementary Table 2 for a list of the studies with descriptions of self-trackers (the people) and self-tracking (the activity).

The following sections elaborate on these three levels of agency, illustrating how self-tracking practices vary from externally governed data collection to self-authored knowledge production.

#### 3.1.1 Low levels of agency

In total, 17 articles were identified as low agency, where 3 describe self-trackers (the people) and 14 describe self-tracking (the activity). Across these low agency studies, participants performed only the data-collection part of Observing themselves, typically by wearing devices or using apps, and did not participate in defining relevant phenomena. Formulating the research question (Questioning) was consistently done by the investigators, not by the users. Similarly, data analysis and visualization (Reasoning) and drawing conclusions (Discovering) about the data was carried out by researchers. In summary, the common pattern is that in these studies users are treated as data-collectors that do not initiate the research or interpret the data. Also analysis and hypothesis-testing are in the researchers' hands.

##### 3.1.1.1 The people

In this group of articles, self-trackers (the people) are portrayed as handing over judgment to devices, folding their lives around metrics, and internalizing optimization norms rather than defining their own aims (26–28). They are described as outsourcing attentiveness and decision-making to devices and data, and aligning behavior to what is measurable and normatively valued. All three articles state that a key activity for self-trackers is to share their data with others (26–28).

##### 3.1.1.2 The activity

Across these studies, self-tracking (the activity) represents instrumental observation: participants collect personal data to supply standardized inputs for researchers rather than to explore personal questions (29–42). The act of observing remains passive: individuals serve as “sensor platforms,” while meaning-making and reasoning are transferred to scientific analysis, reflecting conventional rather than personal science.

#### 3.1.2 Mixed levels of agency

In total, 10 articles were identified as mixed levels of agency, where 7 describe self-trackers (the people) and 3 describe self-tracking (the activity), see Supplementary Table 2 for a list of the studies with brief descriptions. In all these articles, we see higher levels of agency than in the former group, meaning that here, the individuals do more than merely collect data but they are not fully engaging in personal science.

##### 3.1.2.1 The people

Here, self-trackers emerge as negotiators who engage actively with their data but not entirely on their own terms (43–47). These articles share a view of agency as situated, shifting, and co-produced between people, technologies, and sociocultural contexts. Self-tracking becomes a negotiation with one's digital reflection: numbers prompt reflection and change while applying an optimization logic. Knowledge arises as a hybrid—data gain meaning only through personal interpretation. People and technologies co-evolve in a “laboratory of the self,” using “data doubles,” where curiosity and experimentation alternate with fatigue (48, 49). Devices both expand and limit awareness, shaping what feels improvable. Taken together, these studies describe self-trackers' practice as a hybrid of autonomy and dependence. Participants can be active, interpretive, and inventive, but their agency is often mediated by devices, interfaces, social validation, and predictive algorithms. Technologies both enable reflection and structure behavior; knowledge is produced through the interplay of metrics and meaning-making. In these articles, sharing of data is mentioned (47, 49) but more frequently mentioned is sharing of methods, experiences, and learnings (44, 45, 47–49). In one article, it is even mentioned that the person explicitly does not share their data (48).

##### 3.1.2.2 The activity

Across these three articles, self-tracking is depicted as a complex intersection of technology, identity, and self-experimentation (50–52). They illustrate how self-tracking can be understood and interpreted through cultural or theoretical lenses. Here, the logic of personal science, to use one's own data to answer one's own questions, is absent. The authors describe or critique self-tracking practices using their own experiences from self-tracking but without formulating purposeful, self-owned questions or conducting systematic observation and reasoning. Data are collected incidentally or represented abstractly, and discoveries concern cultural meaning rather than personal understanding.

#### 3.1.3 High levels of agency

In total, 19 articles were identified as high agency, where 10 describe self-trackers (the people) and 9 describe self-tracking (the activity), see Supplementary Table 2 for a list of the studies with brief descriptions. In these 19 articles, the full cycle of self-directed inquiry is visible in practice, as an identifiable pattern of who poses the question, who designs the observation, and who interprets the findings: ***Questioning***: Each case begins with a personally meaningful inquiry, often emerging from lived observation—e.g., “Why do I feel sad at certain times?” or “Does my diet cause my afternoon tiredness?” These questions are self-initiated, motivated by curiosity about one's own patterns rather than external research agendas. ***Observing***: Participants engage in systematic, self-directed data collection, using apps, sensors, or manual logs to measure variables like mood, sleep, blood pressure, and blood glucose. The observations are guided by self-defined goals and often refined through iterative methodological reflection, reflecting awareness of reliability and validity. ***Reasoning***: Reasoning occurs through visualization, correlation analysis, and hypothesis testing using tools (e.g., spreadsheets). Self-trackers interpret patterns and sometimes verify findings against scientific literature, blending experiential and scientific reasoning in a personalized, methodically controlled way. ***Discovering***: Insights are integrated into daily life and sometimes shared, for example within a community or group, online or in-person. Explanations take the form of applied self-understanding, such as dietary changes, mood regulation, or medication adjustments, and articulate what the individual has learned about themselves.

##### 3.1.3.1 The people

The articles describing self-trackers of high agency show a clear, shared picture: self-trackers are described as active protagonists who define their own questions, adapt tools to fit those questions, and treat observations and data as prompts for reflection rather than as strict decisions (12, 53–61). Conceptually, self-trackers are portrayed in several, complementary ways: as practitioners of personal science (individuals conducting n-of-1 inquiries to answer life-relevant questions), soft resistance (reclaiming data meaning from institutional categories), mindful/narrative practice (turning metrics into stories that make subjective life legible), a historical continuity with diary-based self-governance, and, from a design lens, the data multiple (accepting that one person's data can sustain many valid interpretations).

Motivations cluster around pragmatic self-improvement (health, sleep, productivity), epistemic curiosity (learning what really works “for me”), and an explicit desire for autonomy—ownership of goals, data, and interpretation. Across the set, technology is a means, not a master: users mix commercial apps with DIY hacks, open platforms, and manual logging; they customize categories, question black-box scores, and sometimes build their own data acquisition instruments or algorithms. The common rule is that *each person remains the final arbiter of their data's meaning*, with peers serving as sounding boards, not authorities. Knowledge is situated, actionable, and provisional (“knowledge-in-the-making”) yet often rigorous enough to yield self-expertise. Limits and tensions are acknowledged—biases of anecdote, uneven tool quality, privacy/ownership concerns, market logics—but the through-line is unmistakable: in high-agency portrayals, self-tracking is less a story of compliance than of craft—iterative, interpretive, and frequently communal work that relocates meaningful authority about the self to the self. Also in the high-agency articles, sharing of data is mentioned (12, 53, 57, 58, 61) and, similarly to the mixed-agency group, sharing of methods, learnings, and experiences is more frequently used. One article uses the term “extreme citizen science” when referring to self-trackers (57) and one article refers to self-trackers as “extreme users” (54).

##### 3.1.3.2 The activity

Across nine articles relating to the activity of self-tracking, we see a consistent pattern of personal science in action, and in all articles, the person doing the tracking is also an author to the scientific article (62–70). All self-trackers began with Questioning: a personal question or goal that was inherently meaningful to them, not one assigned by a traditional researcher. This questioning took many forms: a desire to alleviate a symptom or improve health, e.g. a patient exploring effects of antidepressants on sleep (62), individuals curious about their cholesterol variability (67), or an urge to optimize some aspect of life (65, 68). In each case, the motivation came from the individual—they *owned* the question. Notably, these questions were often distinct from formal scientific hypotheses in their personal framing. For instance, a formal study might ask “Does nicotine reduce dyskinesia in Parkinson's patients?”, whereas the question in the included study was “Will nicotine help *me* manage *my* dyskinesia?” (63)—a subtle but crucial difference that makes it *personal science*.

Once the question was posed, all cases moved into Observing. This was universally self-directed data collection, tailored to the question and the person's life. The articles illustrate how personal scientists can integrate measurement into daily routines. Several leveraged consumer health tech: wearable devices [Fitbit (70), EEG headband (65), continuous glucose monitor (64), portable pollution sensor (69), home lipid analyzers (67)] were common. Importantly, the self-trackers themselves performed the measurements or triggered the data logging, grounding the data in real-life conditions rather than a lab. This often required extra effort or skill: one self-tracker had to write custom software to export his smartwatch data for analysis (66), and the diabetes family manually kept thousands of log entries over years (64). Such dedication highlights a key trait of genuine personal science—the individual is willing to be both the experimenter and the subject, collecting rich data about themselves in pursuit of an answer.

After gathering data, all cases engaged in Reasoning and analysis of their personal data. The complexity of analysis varied, but each person (often with collaborators or tools) sought to interpret what the observations meant with respect to their question. Many employed straightforward visualization and pattern recognition: for example, in a case with a diabetic child, the family saw *gaps* in readings during school hours once they plotted continuous data, directly leading to the insight that their routine led to blind spots (64). In a father–daughter Fitbit study, they computed correlations and trends over time, discovering synchronized patterns and a decline in the teen's steps as she aged (70). In a collaborative QS blood lipid project, each participant effectively performed an N-of-1 analysis: Did their lipids change when expected? Did they cross risk thresholds? Some participants did indeed find dramatic intra-day swings that they would have missed with standard testing (67). A hallmark of personal science reasoning is that *the analysis is tightly focused on actionable insight for the individual*. For instance, a personal productivity experiment sifted through multi-stream data to pinpoint factors influencing *their own* output, leading him to identify specific distractions to minimize (68). Likewise, a self-tracker examined his meditation logs and EEG feedback to conclude that using the device was helping him sustain meditation, an insight directly useful for his practice (65). In several cases, blending subjective and objective data was key. This integration of quantitative and qualitative data is a common pattern in personal science reasoning, leveraging the fact that the self-tracker is also the expert and can interpret results using contextual knowledge.

Finally, every case concluded with a Discovering phase, though the forms varied. All were presented in peer-reviewed publications—effectively translating personal findings into generalizable knowledge or at least documented case reports. The father–daughter team distilled their 5-year endeavor into recommendations for pediatricians and device makers, as well as proof that teens can be engaged in long-term tracking (70). Several projects also presented their findings in the QS community before or alongside academic publication. Drawing on our own direct experience of QS community practice, we note that the QS meeting culture encourages self-trackers to tell the story of their data and self-tracking process—including setbacks and surprises—in meetup talks or online forums. This communal aspect of Discovering underscores another insight: personal science often has a *social component* where sharing is both motivator and reward. By presenting their learnings, personal scientists validate their work and allow others to learn from or build upon it. Regardless of format, in genuine personal science the act of articulation—“What did I do? How did I do it? What did I learn?”—is treated as an integral part of the process, not an afterthought.

## 4 Discussion

The aim of our study was to assess the utility of a framework for characterizing the epistemic dimension of self-tracking, by using it to classify studies according to the level of agency granted to the individual. In doing so, we also examined how personal science—the participatory, inquiry-driven, high-agency form of self-tracking—has been represented in that literature. Our review of papers revealed that while QS has become an established keyword in academic literature, its use is heterogeneous and often only loosely connected to the practices and ethos of the QS community itself. By applying the personal science framework and categorizing eligible studies according to levels of agency, we identified different epistemic orientations—from low-agency, researcher-driven data collection to high-agency, participant-led inquiry. These differences illuminate how scientific framings of QS can either obscure or highlight its original participatory potential and offer lessons for the development of citizen science for health.

### 4.1 The development of quantified self in scientific literature

The appearance of QS in the literature over time as depicted in Figure 3 seems to follow a pattern similar to many technological and social innovations. It looks like a “hype cycle” (71) moving from early enthusiasm to critique and toward more balanced appreciation. Early narratives of universal empowerment were followed by critiques emphasizing surveillance, data commodification, and neoliberal self-optimization. The peak we found for 2018 in the number of publications using the term was therefore what we expected. The uptick in 2024 could potentially indicate that QS is moving toward the “plateau of productivity” in the hype cycle model (71). It is worth noting that this decline may also partly reflect a shift in terminology rather than a decline in the practices themselves: personal science and related activities continue to be widely practiced, but are increasingly discussed under alternative terms such as self-tracking, personal health informatics, or N-of-1 research. This reinforces the conclusion that our findings should be read in the context of a trend, rather than as an account of all personal science activity in the literature.

The early scientific framing of QS appears to have had a lasting influence on how the term has been understood. One of the first scholars to publish about QS in scientific literature and also one of the most cited researchers in the field is Melanie Swan. To date (Oct 2025), the four articles by her published 2009–2013 included in our review (53, 72–74) have been cited in total more than 1,400 times. Swan's descriptions of QS as a data-driven movement supporting preventive, personalized, and predictive medicine steered subsequent discourse toward optimization and big data paradigms. This primacy effect illustrates how early publications can create a path dependence in emerging fields, including assumptions that later scholarship often inherits. As a result, much of the literature reproduces a view of QS aligned with biomedical or technological perspectives, rather than the reflexive, community-based, and experiential practices found within the QS movement itself.

### 4.2 Level of agency as an analytical lens

Using our four-phase personal science framework (Questioning–Observing–Reasoning–Discovering) to assess the 46 articles included in our full analysis, we were able to categorize papers based on the level of agency of the individual research subjects. In low-agency articles, participants are treated primarily as data sources (or sensor platforms), contributing observations but not participating in defining questions or interpreting results. In mixed-agency articles, individuals may engage with data and technology in reflexive ways but remain constrained by external goals or platform logics. Only in high-agency articles do we see the full cycle of personal science, where individuals define their own questions, collect and analyze their data, and articulate what they have learned. We found it noteworthy that among the low-agency articles describing the people doing self-tracking, data sharing was described as a key aspect whereas in articles with higher levels of agency, sharing of methods, experiences, and learnings were more frequently mentioned.

These differences suggest that our analytical framework of epistemic agency is useful for distinguishing between “self-tracking as only measurement” and “self-tracking as an element of knowledge creation with epistemic agency.” In the context of QS, the key distinction lies not in the use of self-tracking technology per se but in ***who owns*** ***the question***. When the individual defines the problem and interprets the findings, self-tracking becomes personal science; when researchers or technologies determine what counts as meaningful, agency diminishes and self-tracking collapses into mere data production.

Two features—the identity of researcher and subject, and the aim of self-knowledge—distinguish personal science from the qualitative research tradition, which similarly treats subjective experience as a legitimate source of knowledge. Qualitative research uses subjective interpretation as a methodological choice, in service of conceptual understanding that transfers beyond the individual participant. In personal science, combining subjective and objective data is not a methodological choice but a structural feature: because the researcher and the subject are the same person, subjective experience is not an alternative to measurement but a necessary part of the evidence, and unlike qualitative research, the persons contextual knowledge can be applied in interpretation of both types of data. The purpose differs as well—not conceptual generalization but actionable self-knowledge. Personal science is therefore not a variant of qualitative inquiry but a form of empirical practice to be judged by its own standards of relevance.

Taken together, these findings highlight that the scientific literature on QS only partially captures the phenomenon's participatory core. Articles labeled “quantified self” may reflect anything from traditional observational studies to high agency citizen science for health; that is, personal science.

### 4.3 Relevance for citizen science

A comparison between personal science and citizen science as typically understood highlights both shared values and crucial differences. Both movements democratize inquiry and expand the range of who can generate and validate knowledge. However, whereas traditional citizen science typically informs public knowledge, focusing on collective phenomena such as environmental monitoring or epidemiological trends, personal science informs self-knowledge, concerned with the lived body and subjective experience. The former seeks generalizable knowledge; the latter prioritizes actionable understanding for the individual. Despite this distinction, both approaches contribute to what might be termed “epistemic capacity,” meaning the ability of individuals and communities to use empirical reasoning to make sense of their world. Personal science represents a dimension of citizen science for health where the individual is both subject and scientist. Supporting personal science may therefore contribute not only to individual empowerment but to the broader ecosystem of participatory knowledge production—a hypothesis that future empirical research could test.

The three-level, agency-based categorization we developed offers a straightforward way to assess epistemic agency, and it could be applied more broadly to citizen science for health projects, patient-led research, or participatory health initiatives, providing a way to gauge the depth of participation and control at each stage of the knowledge cycle. This overlaps with other participation models in citizen science, where higher levels of autonomy correspond to deeper forms of engagement and learning. Our levels of agency roughly map onto the participatory levels defined by Den Broeder et al. (16): our low-agency articles, in which self-trackers are treated as sensor platforms, correspond to the level of crowd sourcing while our high-agency level corresponds to their highest participatory level, which Haklay terms “extreme citizen science” (17). But we should note that in only two of our 19 high-agency articles were personal scientists described as “extreme” (54, 57), and both those were written from outside the QS community. Den Broeder et al. note that “extreme” need not mean rare (16). Because this label is not used by practitioners themselves, and its connotation risks presenting a routine practice as an outlier, we suggest “personal science” and “personal scientists” as more accurate descriptors.

### 4.4 Conclusions

Realizing the potential of personal science within citizen science requires infrastructure, recognition, and collaboration. Unlike institutional research, personal science often operates outside formal ethical oversight, yet it raises ethical concerns regarding data security, validity, and potential harm. Institutions could help by establishing education and training programs that link self-trackers, clinicians, and researchers. Programs in health literacy and patient education could incorporate personal science methods, helping individuals ask empirical questions about their own health and evaluate data critically. Ethical guidelines and community consultation mechanisms, similar to those emerging in citizen science, could add confidence that self-directed inquiry is empowering and responsible. Moreover, personal science could benefit from infrastructure for knowledge sharing among peers, including documentation and review.

Extending citizen participation in health research from merely contributing data to cultivating ***thinking scientifically about oneself*** can help foster an informed, empowered public capable of contributing to collective wellbeing.

### 4.5 Limitations

We know that not all papers that report on self-tracking or quantified self have been found in our literature search. Some specific contributions by academic participants in the Quantified Self community have not been captured by our search because they did not use self-tracking and quantified self as keywords. These “known unknowns” (75–78) all share a feature in common: they are first person accounts of the use and translation of personal discoveries using self-tracking into a general set of methods for aiding patients and advancing science. Knowing this, we still decided not to change our search strategy. Despite missing some papers that represent personal science, our assessment is that this number is small and the missing data have limited effect on our analysis and our conclusions.

Our positionality as insiders to the QS community may have shaped our interpretations in specific ways. We are inclined to recognize and value high-agency practices in the literature, which may mean we were more sensitive to their presence than an outside reviewer would be. Conversely, we may have been more critical of framings that we perceived as misrepresenting the QS community's self-understanding, particularly the sociological critique literature and the digital health optimization framing. We have sought to address this by presenting our findings transparently, including cases where the literature's framing resists or complicates our own framework, and by making our analytical categories and decision rules explicit and available for scrutiny. We encourage readers to evaluate our interpretations in light of this positionality.
