# Supplementary material for: Epistemic agency in self-tracking: a conceptual literature review of quantified self as citizen science for health
Source: Front Public Health. 2026 Aug 18;14:1736850. doi: 10.3389/fpubh.2026.1736850 (PMC13530006; doi:10.3389/fpubh.2026.1736850)
Supplement: Supplementary file 1 [file Data_Sheet_1.pdf]

Supplementary table 1. Information for the included 241 articles listed in chronological order.

Article information
Swan, M. Emerging Patient-Driven Health Care Models: An Examination of Health Social Networks, Consumer Personalized Medicine and Quantified Self-Tracking. INTERNATIONAL JOURNAL OF ENVIRONMENTAL RESEARCH AND PUBLIC HEALTH. 2009
Swan, M. Health 2050: The Realization of Personalized Medicine through Crowdsourcing, the Quantified Self, and the Participatory Biocitizen. JOURNAL OF PERSONALIZED MEDICINE. 2012
Swan, M. Crowdsourced Health Research Studies: An Important Emerging Complement to Clinical Trials in the Public Health Research Ecosystem. JOURNAL OF MEDICAL INTERNET RESEARCH. 2012
Swan, M. THE QUANTIFIED SELF: Fundamental Disruption in Big Data Science and Biological Discovery. BIG DATA. 2013
Lupton, D. Quantifying the body: monitoring and measuring health in the age of mHealth technologies. CRITICAL PUBLIC HEALTH. 2013
Whitson, JR. Gaming the Quantified Self. SURVEILLANCE & SOCIETY. 2013
Chen, ZY; Lin, M; Chen, FL; Lane, ND; Cardone, G; Wang, R; Li, TX; Chen, YQ; Choudhury, T; Campbell, AT. Unobtrusive Sleep Monitoring using Smartphones. PROCEEDINGS OF THE 2013 7TH INTERNATIONAL CONFERENCE ON PERVASIVE COMPUTING TECHNOLOGIES FOR HEALTHCARE AND WORKSHOPS (PERVASIVEHEALTH 2013). 2013
Barrett, MA; Humblet, O; Hiatt, RA; Adler, NE. BIG DATA AND DISEASE PREVENTION: From Quantified Self to Quantified Communities. BIG DATA. 2013
Bossewitch, J; Sinnreich, A. The end of forgetting: Strategic agency beyond the panopticon. NEW MEDIA & SOCIETY. 2013
Lee, VR; Drake, J. Digital Physical Activity Data Collection and Use by Endurance Runners and Distance Cyclists. TECHNOLOGY KNOWLEDGE AND LEARNING. 2013
Matrix, S. The Chronopolitical Order of Things: Technologies of the Quantified Self in Andrew Niccol's In Time and Michael Anderson's Logan's Run. CRITICAL SURVEY. 2013
Krijnen, D; Riphagen, M; van Hout, M; Gootjes, G. LEARNING TOMORROW: VISUALISING STUDENT AND STAFF PHYSICAL ACTIVITY. EDULEARN13: 5TH INTERNATIONAL CONFERENCE ON EDUCATION AND NEW LEARNING TECHNOLOGIES. 2013
Wolbring G, Leopatra V.. Sensors: views of staff of a disability service organization. J Pers Med. 2013 Feb 22. 2013
Choe, EK; Lee, NB; Lee, B; Pratt, W; Kientz, JA. Understanding Quantified-Selfers' Practices in Collecting and Exploring Personal Data. 32ND ANNUAL ACM CONFERENCE ON HUMAN FACTORS IN COMPUTING SYSTEMS (CHI 2014). 2014
Rooksby, J; Rost, M; Morrison, A; Chalmers, M. Personal Tracking as Lived Informatics. 32ND ANNUAL ACM CONFERENCE ON HUMAN FACTORS IN COMPUTING SYSTEMS (CHI 2014). 2014
Ruckenstein, M. Visualized and Interacted Life: Personal Analytics and Engagements with Data Doubles. SOCIETIES. 2014
Nafus, D; Sherman, J. This One Does Not Go Up to 11: The Quantified Self Movement as an Alternative Big Data Practice. INTERNATIONAL JOURNAL OF COMMUNICATION. 2014
Till, C. Exercise as Labour: Quantified Self and the Transformation of Exercise into Labour. SOCIETIES. 2014
Meadow, JF; Altrichter, AE; Green, JL. Mobile phones carry the personal microbiome of their owners. PEERJ. 2014
Reigeluth, T. Why data is not enough: Digital traces as control of self and self-control. SURVEILLANCE & SOCIETY. 2014

**Article information**
Harrison, D; Marshall, P; Berthouze, N; Bird, J. Tracking Physical Activity: Problems Related to Running Longitudinal Studies with Commercial Devices. PROCEEDINGS OF THE 2014 ACM INTERNATIONAL JOINT CONFERENCE ON PERVASIVE AND UBIQUITOUS COMPUTING (UBICOMP'14 ADJUNCT). 2014
Hirsch, JA; James, P; Robinson, JRM; Eastman, KM; Conley, KD; Evenson, KR; Laden, F. Using MapMyFitness to place physical activity into neighborhood context. FRONTIERS IN PUBLIC HEALTH. 2014
Appelboom, G; LoPresti, M; Reginster, JY; Connolly, ES; Dumont, EPL. The quantified patient: a patient participatory culture. CURRENT MEDICAL RESEARCH AND OPINION. 2014
Beauchet, O; Launay, CP; Merjagnan, C; Kabeshova, A; Annweiler, C. Quantified Self and Comprehensive Geriatric Assessment: Older Adults Are Able to Evaluate Their Own Health and Functional Status. PLOS ONE. 2014
Kim, J. A Qualitative Analysis of User Experiences With a Self-Tracker for Activity, Sleep, and Diet. INTERACTIVE JOURNAL OF MEDICAL RESEARCH. 2014
Jethani, S. Mediating the body: technology, politics and epistemologies of self. COMMUNICATION POLITICS & CULTURE. 2014
Lee, VR; Briggs, M. Lessons learned from an initial effort to bring a Quantified Self meetup experience to a new demographic. PROCEEDINGS OF THE 2014 ACM INTERNATIONAL JOINT CONFERENCE ON PERVASIVE AND UBIQUITOUS COMPUTING (UBICOMP'14 ADJUNCT). 2014
Mayor, E; Gamaiunova, L. Using wearable technology for psychophysiological experiments. Gender roles and cognitive appraisal impact cardiac response to socio-evaluative stress. 2014 EAI 4TH INTERNATIONAL CONFERENCE ON WIRELESS MOBILE COMMUNICATION AND HEALTHCARE (MOBIHEALTH). 2014
De Filippi, P. Big data, big responsibilities. INTERNET POLICY REVIEW. 2014
Rettberg, JW. Quantified Selves. SEEING OURSELVES THROUGH TECHNOLOGY: HOW WE USE SELFIES, BLOGS AND WEARABLE DEVICES TO SEE AND SHAPE OURSELVES. 2014
Ancker, JS; Witteman, HO; Hafeez, B; Provencher, T; Van de Graaf, M; Wei, E. 'You Get Reminded You're a Sick Person: Personal Data Tracking and Patients With Multiple Chronic Conditions. JOURNAL OF MEDICAL INTERNET RESEARCH. 2015
Choe, EK; Lee, B; Kay, M; Pratt, W; Kientz, JA. SleepTight: Low-burden, Self-monitoring Technology for Capturing and Reflecting on Sleep Behaviors. PROCEEDINGS OF THE 2015 ACM INTERNATIONAL JOINT CONFERENCE ON PERVASIVE AND UBIQUITOUS COMPUTING (UBICOMP 2015). 2015
Harrison, D; Marshall, P; Bianchi-Berthouze, N; Bird, J. Activity Tracking: Barriers, Workarounds and Customisation. PROCEEDINGS OF THE 2015 ACM INTERNATIONAL JOINT CONFERENCE ON PERVASIVE AND UBIQUITOUS COMPUTING (UBICOMP 2015). 2015
Chung, CF; Cook, J; Bales, E; Zia, J; Munson, SA. More Than Telemonitoring: Health Provider Use and Nonuse of Life-Log Data in Irritable Bowel Syndrome and Weight Management. JOURNAL OF MEDICAL INTERNET RESEARCH. 2015
Hood, L; Lovejoy, JC; Price, ND. Integrating big data and actionable health coaching to optimize wellness. BMC MEDICINE. 2015
Fawcett, T. Mining the Quantified Self: Personal Knowledge Discovery as a Challenge for Data Science. BIG DATA. 2015
Janssen, CP; Gould, SJJ; Li, SYW; Brumby, DR; Cox, AL. Integrating knowledge of multitasking and interruptions across different perspectives and research methods. INTERNATIONAL JOURNAL OF HUMAN-COMPUTER STUDIES. 2015
Majmudar, MD; Colucci, LA; Landman, AB. The quantified patient of the future: Opportunities and challenges. HEALTHCARE-THE JOURNAL OF DELIVERY SCIENCE AND INNOVATION. 2015

**Article information**
Felipe, S; Singh, A; Bradley, C; Williams, ACD; Bianchi-Berthouze, N. Roles for Personal Informatics in Chronic Pain. PROCEEDINGS OF THE 2015 9TH INTERNATIONAL CONFERENCE ON PERVASIVE COMPUTING TECHNOLOGIES FOR HEALTHCARE (PERVASIVEHEALTH). 2015
Park, SY; Chen, YA. Individual and Social Recognition: Challenges and Opportunities in Migraine Management. PROCEEDINGS OF THE 2015 ACM INTERNATIONAL CONFERENCE ON COMPUTER-SUPPORTED COOPERATIVE WORK AND SOCIAL COMPUTING (CSCW'15). 2015
Haddadi, H; Ofli, F; Mejova, Y; Weber, I; Srivastava, J. 360° Quantified Self. 2015 IEEE INTERNATIONAL CONFERENCE ON HEALTHCARE INFORMATICS (ICHI 2015). 2015
Gimbert, C; Lapointe, FJ. Self-tracking the microbiome: where do we go from here?. MICROBIOME. 2015
Thornquist, E; Kirkengen, AL. The quantified self: closing the gap between general knowledge and particular case?. JOURNAL OF EVALUATION IN CLINICAL PRACTICE. 2015
Altenhoff, B; Vaigneur, H; Caine, K. One Step Forward, Two Steps Back: The Key to Wearables in the Field is the App. PROCEEDINGS OF THE 2015 9TH INTERNATIONAL CONFERENCE ON PERVASIVE COMPUTING TECHNOLOGIES FOR HEALTHCARE (PERVASIVEHEALTH). 2015
Brabazon, T. Digital fitness: Self-monitored fitness and the commodification of movement. COMMUNICATION POLITICS & CULTURE. 2015
Guo, L. Nudge: Better Quantified-Self with Context-Aware and Proactive Services. CIT/IUCC/DASC/PICOM 2015 IEEE INTERNATIONAL CONFERENCE ON COMPUTER AND INFORMATION TECHNOLOGY - UBIQUITOUS COMPUTING AND COMMUNICATIONS - DEPENDABLE, AUTONOMIC AND SECURE COMPUTING - PERVASIVE INTELLIGENCE AND COMPUTING. 2015
Dontje ML, de Groot M, Lengton RR, van der Schans CP, Krijnen WP.. Measuring steps with the Fitbit activity tracker: an inter-device reliability study. J Med Eng Technol. 2015. 2015
Kooiman TJ, Dontje ML, Sprenger SR, Krijnen WP, van der Schans CP, de Groot M.. Reliability and validity of ten consumer activity trackers. BMC Sports Sci Med Rehabil. 2015 Oct 12. 2015
Moore, P; Robinson, A. The quantified self: What counts in the neoliberal workplace. NEW MEDIA & SOCIETY. 2016
Lupton, D. The diverse domains of quantified selves: self-tracking modes and dataveillance. ECONOMY AND SOCIETY. 2016
Kostkova, P; Brewer, H; de Lusignan, S; Fottrell, E; Goldacre, B; Hart, G; Koczan, P; Knight, P; Marsolier, C; McKendry, RA; Ross, E; Sasse, A; Sullivan, R; Chaytor, S; Stevenson, O; Velho, R; Tooke, J. Who Owns the Data? Open Data for Healthcare. FRONTIERS IN PUBLIC HEALTH. 2016
Rapp, A; Cena, F. Personal informatics for everyday life: How users without prior self-tracking experience engage with personal data. INTERNATIONAL JOURNAL OF HUMAN-COMPUTER STUDIES. 2016
Bull, S; Kay, J. SMILI (sic): a Framework for Interfaces to Learning Data in Open Learner Models, Learning Analytics and Related Fields. INTERNATIONAL JOURNAL OF ARTIFICIAL INTELLIGENCE IN EDUCATION. 2016
Gilmore, JN. Everywear: The quantified self and wearable fitness technologies. NEW MEDIA & SOCIETY. 2016
Stragier, J; Vanden Abeele, M; Mechant, P; De Marez, L. Understanding persistence in the use of Online Fitness Communities: Comparing novice and experienced users. COMPUTERS IN HUMAN BEHAVIOR. 2016
West, P; Giordano, R; Van Kleek, M; Shadbolt, N. The Quantified Patient in the Doctor's Office: Challenges & Opportunities. 34TH ANNUAL CHI CONFERENCE ON HUMAN FACTORS IN COMPUTING SYSTEMS, CHI 2016. 2016
Barta, K; Neff, G. Technologies for Sharing: lessons from Quantified Self about the political economy of platforms. INFORMATION COMMUNICATION & SOCIETY. 2016

Article information
Lanzing, M. The transparent self. ETHICS AND INFORMATION TECHNOLOGY. 2016
Lee, VR; Drake, JR; Thayne, JL. Appropriating Quantified Self Technologies to Support Elementary Statistical Teaching and Learning. IEEE TRANSACTIONS ON LEARNING TECHNOLOGIES. 2016
Elsden, C; Durrant, AC; Kirk, DS. It's just my history, isn't it?: Understanding Smart Journaling Practices. 34TH ANNUAL CHI CONFERENCE ON HUMAN FACTORS IN COMPUTING SYSTEMS, CHI 2016. 2016
Ruiz, S; Charleer, S; Urretavizcaya, M; Klerkx, J; Fernández-Castro, I; Duval, E. Supporting learning by considering emotions: Tracking and Visualization. A case study. LAK '16 CONFERENCE PROCEEDINGS: THE SIXTH INTERNATIONAL LEARNING ANALYTICS & KNOWLEDGE CONFERENCE,. 2016
Weber, I; Achananuparp, P. INSIGHTS FROM MACHINE-LEARNED DIET SUCCESS PREDICTION. PACIFIC SYMPOSIUM ON BIOCOMPUTING 2016. 2016
Teston, C. Rhetoric, Precarity, and mHealth Technologies. RHETORIC SOCIETY QUARTERLY. 2016
Wang, YF; Weber, I; Mitra, P. Quantified Self Meets Social Media: Sharing of Weight Updates on Twitter. DH'16: PROCEEDINGS OF THE 2016 DIGITAL HEALTH CONFERENCE. 2016
Gollamudi, SS; Topol, EJ; Wineinger, NE. A framework for smartphone-enabled, patient-generated health data analysis. PEERJ. 2016
Verdezoto, N; Grönvall, E. On preventive blood pressure self-monitoring at home. COGNITION TECHNOLOGY & WORK. 2016
Clinch, S; Davies, N; Mikusz, M; Metzger, P; Langheinrich, M; Schmidt, A; Ward, G. Collecting Shared Experiences through Lifelogging: Lessons Learned. IEEE PERVASIVE COMPUTING. 2016
Visuri, A; Sarsenbayeva, Z; Goncalves, J; Karapanos, E; Jones, S. Impact of Mood Changes on Application Selection. UBICOMP'16 ADJUNCT: PROCEEDINGS OF THE 2016 ACM INTERNATIONAL JOINT CONFERENCE ON PERVASIVE AND UBIQUITOUS COMPUTING. 2016
Marcengo, A; Rapp, A; Cena, F; Geymonat, M. The Falsified Self: Complexities in Personal Data Collection. UNIVERSAL ACCESS IN HUMAN-COMPUTER INTERACTION; METHODS, TECHNIQUES, AND BEST PRACTICES, PT I. 2016
Dietrich, M; van Laerhoven, K. An Interdisciplinary Approach on the Mediating Character of Technologies for Recognizing Human Activity. PHILOSOPHIES. 2016
Chowdhury, S; Ferdous, MS; Jose, JM. A User-Study Examining Visualization of Lifelogs. 2016 14TH INTERNATIONAL WORKSHOP ON CONTENT-BASED MULTIMEDIA INDEXING (CBMI). 2016
Hagood, D; Ching, CC; Schaefer, S. Integrating Physical Activity Data in Videogames with User-Centered Dashboards. LAK '16 CONFERENCE PROCEEDINGS: THE SIXTH INTERNATIONAL LEARNING ANALYTICS & KNOWLEDGE CONFERENCE,. 2016
Müllner, R. Self-Improvement In and Through Sports: Cultural-Historical Perspectives. INTERNATIONAL JOURNAL OF THE HISTORY OF SPORT. 2016
Bækgaard, P; Petersen, MK; Larsen, JE. Assessing Levels of Attention Using Low Cost Eye Tracking. UNIVERSAL ACCESS IN HUMAN-COMPUTER INTERACTION; METHODS, TECHNIQUES, AND BEST PRACTICES, PT I. 2016
Ishimaru, S; Dingier, T; Kunze, K; Kise, K; Dengel, A. Reading Interventions - Tracking Reading State and Designing Interventions. UBICOMP'16 ADJUNCT: PROCEEDINGS OF THE 2016 ACM INTERNATIONAL JOINT CONFERENCE ON PERVASIVE AND UBIQUITOUS COMPUTING. 2016
O'Neill, S. Stripe Painting: A Method of Expressing the Experience of Cycling Through 'Quantified Self' Data Visualisation. UBICOMP'16 ADJUNCT: PROCEEDINGS OF THE 2016 ACM INTERNATIONAL JOINT CONFERENCE ON PERVASIVE AND UBIQUITOUS COMPUTING. 2016

Article information
Liang, ZL; Nagata, Y; Martell, MAC; Nishimura, T. Nurturing Wearable and mHealth Technologies for Self-Care: Mindset, Tool Set and Skill Set. 2016 IEEE 18TH INTERNATIONAL CONFERENCE ON E-HEALTH NETWORKING, APPLICATIONS AND SERVICES (HEALTHCOM). 2016
Magnuson V, Wang Y, Schork N.. Normalizing sleep quality disturbed by psychiatric polypharmacy: a single patient open trial (SPOT). F1000Res. 2016 Feb 3. 2016
Ruckenstein, M; Schüll, ND. The Datafication of Health. ANNUAL REVIEW OF ANTHROPOLOGY, VOL 46. 2017
Ajana, B. Digital health and the biopolitics of the Quantified Self. DIGITAL HEALTH. 2017
Sharon, T; Zandbergen, D. From data fetishism to quantifying selves: Self-tracking practices and the other values of data. NEW MEDIA & SOCIETY. 2017
Fokkema, T; Kooiman, TJM; Krijnen, WP; Van der Schans, CP; De Groot, M. Reliability and Validity of Ten Consumer Activity Trackers Depend on Walking Speed. MEDICINE & SCIENCE IN SPORTS & EXERCISE. 2017
Ruckenstein, M; Pantzar, M. Beyond the Quantified Self: Thematic exploration of a dataistic paradigm. NEW MEDIA & SOCIETY. 2017
Fotopoulou, A; O'Riordan, K. Training to self-care: fitness tracking, biopedagogy and the healthy consumer. HEALTH SOCIOLOGY REVIEW. 2017
Kelley, C; Lee, B; Wilcox, L. Self-tracking for Mental Wellness: Understanding Expert Perspectives and Student Experiences. PROCEEDINGS OF THE 2017 ACM SIGCHI CONFERENCE ON HUMAN FACTORS IN COMPUTING SYSTEMS (CHI'17). 2017
Shin, DH; Biocca, F. Health experience model of personal informatics: The case of a quantified self. COMPUTERS IN HUMAN BEHAVIOR. 2017
Baron, KG; Abbott, S; Jao, N; Manalo, N; Mullen, R. Orthosomnia: Are Some Patients Taking the Quantified Self Too Far?. JOURNAL OF CLINICAL SLEEP MEDICINE. 2017
Khot, RA; Aggarwal, D; Pennings, R; Hjorth, L; Mueller, F. EdiPulse: Investigating a Playful Approach to Self-monitoring through 3D Printed Chocolate Treats. PROCEEDINGS OF THE 2017 ACM SIGCHI CONFERENCE ON HUMAN FACTORS IN COMPUTING SYSTEMS (CHI'17). 2017
Bolaños, M; Dimiccoli, M; Radeva, P. Toward Storytelling From Visual Lifelogging: An Overview. IEEE TRANSACTIONS ON HUMAN-MACHINE SYSTEMS. 2017
Ravichandran, R; Sien, SW; Patel, SN; Kientz, JA; Pina, LR. Making Sense of Sleep Sensors: How Sleep Sensing Technologies Support and Undermine Sleep Health. PROCEEDINGS OF THE 2017 ACM SIGCHI CONFERENCE ON HUMAN FACTORS IN COMPUTING SYSTEMS (CHI'17). 2017
Eikey, EV; Reddy, MC. It's Definitely Been a Journey: A Qualitative Study on How Women with Eating Disorders Use Weight Loss Apps. PROCEEDINGS OF THE 2017 ACM SIGCHI CONFERENCE ON HUMAN FACTORS IN COMPUTING SYSTEMS (CHI'17). 2017
Moore, P; Piwek, L. Regulating wellbeing in the brave new quantified workplace. EMPLOYEE RELATIONS. 2017
Wexler, A. The Social Context of Do-It-Yourself Brain Stimulation: Neurohackers, Biohackers, and Lifehackers. FRONTIERS IN HUMAN NEUROSCIENCE. 2017
Smith, GJD; Vonthethoff, B. Health by numbers? Exploring the practice and experience of datafied health. HEALTH SOCIOLOGY REVIEW. 2017
Barassi, V. BabyVeillance? Expecting Parents, Online Surveillance and the Cultural Specificity of Pregnancy Apps. SOCIAL MEDIA + SOCIETY. 2017
West, P; Van Kleek, M; Giordano, R; Weal, M; Shadbolt, N. Information Quality Challenges of Patient-Generated Data in Clinical Practice. FRONTIERS IN PUBLIC HEALTH. 2017
Malgieri, G; Comandé, G. Sensitive-by-distance: quasi-health data in the algorithmic era. INFORMATION & COMMUNICATIONS TECHNOLOGY LAW. 2017

Article information
O'Neill, C. Taylorism, the European Science of Work, and the Quantified Self at Work. SCIENCE TECHNOLOGY & HUMAN VALUES. 2017
Fernandez-Luque, L; Singh, M; Ofli, F; Mejova, YA; Weber, I; Aupetit, M; Jreige, SK; Elmagarmid, A; Srivastava, J; Ahmedna, M. Implementing 360° Quantified Self for childhood obesity: feasibility study and experiences from a weight loss camp in Qatar. BMC MEDICAL INFORMATICS AND DECISION MAKING. 2017
Pettinico, G; Milne, GR. Living by the numbers: understanding the quantification effect. JOURNAL OF CONSUMER MARKETING. 2017
Kleinpeter, E. Four Ethical Issues of E-Health. IRBM. 2017
Riggare, S; Unruh, KT; Sturr, J; Domingos, J; Stamford, JA; Svenningsson, P; Hägglund, M. Patient-driven N-of-1 in Parkinson's Disease Lessons Learned from a Placebo-controlled Study of the Effect of Nicotine on Dyskinesia. METHODS OF INFORMATION IN MEDICINE. 2017
Meng, JB; Peng, W; Shin, SY; Chung, M. Online Self-Tracking Groups to Increase Fruit and Vegetable Intake: A Small-Scale Study on Mechanisms of Group Effect on Behavior Change. JOURNAL OF MEDICAL INTERNET RESEARCH. 2017
Dolejsová, M; Kera, D. Soylent Diet Self-Experimentation: Design Challenges in Extreme Citizen Science Projects. CSCW'17: PROCEEDINGS OF THE 2017 ACM CONFERENCE ON COMPUTER SUPPORTED COOPERATIVE WORK AND SOCIAL COMPUTING. 2017
O'Neill, C. Haptic media and the cultural techniques of touch: The sphygmograph, photoplethysmography and the Apple Watch. NEW MEDIA & SOCIETY. 2017
Lee, V; Thurston, T; Thurston, C. A Comparison of Discovered Regularities in Blood Glucose Readings across Two Data Collection Approaches Used with a Type 1 Diabetic Youth. METHODS OF INFORMATION IN MEDICINE. 2017
Iaconesi, S. Interface and Data Biopolitics in the Age of Hyperconnectivity. Implications for Design. DESIGN JOURNAL. 2017
Marcengo, A; Sabena, E; Crea, A. Mindfulness and Asynchronous Neurofeedback: Coping with Mind Wandering. UNIVERSAL ACCESS IN HUMAN-COMPUTER INTERACTION: HUMAN AND TECHNOLOGICAL ENVIRONMENTS, PT III. 2017
Alelyani, S; Ibrahim, A. Would quantified self prevent obesity and diabetes among adults in Saudi Arabia?. 2017 INTERNATIONAL CONFERENCE ON INFORMATICS, HEALTH & TECHNOLOGY (ICIHT). 2017
Olsson, CM. Engagement Issues in Self-Tracking Lessons Learned from User Feedback of Three Major Self-Tracking Services. 2017 IEEE INTERNATIONAL CONFERENCE ON PERVASIVE COMPUTING AND COMMUNICATIONS WORKSHOPS (PERCOM WORKSHOPS). 2017
Sacramento, I; Wanick, V. mHealth and the Digital Cyborg Body: The Running Apps in a Society of Control. MOBILE E-HEALTH. 2017
Nichols, M. IDENTIFYING WITH NUMBERS: A PHILOSOPHICAL AND PSYCHOANALYTICAL READING OF SELF-IDENTIFICATION. STUDIA UNIVERSITATIS BABES-BOLYAI PHILOSOPHIA. 2017
Hedin, B; Claesson, P; Odqvist, P. Visualizing Carbon Footprint from School Meals. 2017 FIFTH IFIP CONFERENCE ON SUSTAINABLE INTERNET AND ICT FOR SUSTAINABILITY (SUSTAINIT 2017). 2017
Hermsen S, Moons J, Kerkhof P, Wiekens C, De Groot M.. Determinants for Sustained Use of an Activity Tracker: Observational Study. JMIR Mhealth Uhealth. 2017 Oct 30. 2017
Kristensen, DB; Ruckenstein, M. Co-evolving with self-tracking technologies. NEW MEDIA & SOCIETY. 2018
Hamari, J; Hassan, L; Dias, A. Gamification, quantified-self or social networking? Matching users' goals with motivational technology. USER MODELING AND USER-ADAPTED INTERACTION. 2018

Article information
Didziokaite, G; Saukko, P; Greiffenhagen, C. The mundane experience of everyday calorie trackers: Beyond the metaphor of Quantified Self. NEW MEDIA & SOCIETY. 2018
Paré, G; Leaver, C; Bourget, C. Diffusion of the Digital Health Self-Tracking Movement in Canada: Results of a National Survey. JOURNAL OF MEDICAL INTERNET RESEARCH. 2018
West, P; Van Kleek, M; Giordano, R; Weal, MJ; Shadbolt, N. Common Barriers to the Use of Patient-Generated Data Across Clinical Settings. PROCEEDINGS OF THE 2018 CHI CONFERENCE ON HUMAN FACTORS IN COMPUTING SYSTEMS (CHI 2018). 2018
Chrisinger, BW; King, AC. Stress experiences in neighborhood and social environments (SENSE): a pilot study to integrate the quantified self with citizen science to improve the built environment and health. INTERNATIONAL JOURNAL OF HEALTH GEOGRAPHICS. 2018
Moore, PV. Tracking Affective Labour for Agility in the Quantified Workplace. BODY & SOCIETY. 2018
Rapp, A; Tirabeni, L. Personal Informatics for Sport: Meaning, Body, and Social Relations in Amateur and Elite Athletes. ACM TRANSACTIONS ON COMPUTER-HUMAN INTERACTION. 2018
Maltseva, K; Lutz, C. A quantum of self: A study of self-quantification and self-disclosure. COMPUTERS IN HUMAN BEHAVIOR. 2018
Danaher, J; Nyholm, S; Earp, BD. The Quantified Relationship. AMERICAN JOURNAL OF BIOETHICS. 2018
Gabriels, K; Moerenhout, T. Exploring Entertainment Medicine and Professionalization of Self-Care: Interview Study Among Doctors on the Potential Effects of Digital Self-Tracking. JOURNAL OF MEDICAL INTERNET RESEARCH. 2018
Steinert, A; Haesner, M; Steinhagen-Thiessen, E. Activity-tracking devices for older adults: comparison and preferences. UNIVERSAL ACCESS IN THE INFORMATION SOCIETY. 2018
Rosen, LD; Carrier, LM; Pedroza, JA; Elias, S; O'Brien, KM; Lozano, J; Kim, K; Cheever, NA; Bentley, J; Ruiz, A. The Role of Executive Functioning and Technological Anxiety (FOMO) in College Course Performance as Mediated by Technology Usage and Multitasking Habits. PSICOLOGIA EDUCATIVA. 2018
Vitak, J; Liao, Y; Kumar, P; Zimmer, M; Kritikos, K. Privacy Attitudes and Data Valuation Among Fitness Tracker Users. TRANSFORMING DIGITAL WORLDS, ICONFERENCE 2018. 2018
Lyall, B; Robards, B. Tool, toy and tutor: Subjective experiences of digital self-tracking. JOURNAL OF SOCIOLOGY. 2018
de Arriba-Pérez, F; Caeiro-Rodríguez, M; Santos-Gago, JM. How do you sleep? Using off the shelf wrist wearables to estimate sleep quality, sleepiness level, chronotype and sleep regularity indicators. JOURNAL OF AMBIENT INTELLIGENCE AND HUMANIZED COMPUTING. 2018
Katz, DS; Price, BA; Holland, S; Dalton, NS. Data, Data Everywhere, and Still Too Hard to Link: Insights from User Interactions with Diabetes Apps. PROCEEDINGS OF THE 2018 CHI CONFERENCE ON HUMAN FACTORS IN COMPUTING SYSTEMS (CHI 2018). 2018
Milward, J; Deluca, P; Drummond, C; Kimergård, A. Developing Typologies of User Engagement With the BRANCH Alcohol-Harm Reduction Smartphone App: Qualitative Study. JMIR MHEALTH AND UHEALTH. 2018
Ajana, B. Introduction: Metric Culture and the Over-examined Life. METRIC CULTURE: ONTOLOGIES OF SELF-TRACKING PRACTICES. 2018
Samerski, S. Individuals on alert: digital epidemiology and the individualization of surveillance. LIFE SCIENCES SOCIETY AND POLICY. 2018
Vesnic-Alujevic, L; Breitegger, M; Pereira, AG. Do-It-Yourself' Healthcare? Quality of Health and Healthcare Through Wearable Sensors. SCIENCE AND ENGINEERING ETHICS. 2018
Rapp, A; Marcengo, A; Buriano, L; Ruffo, G; Lai, M; Cena, F. Designing a personal informatics system for users without experience in self-tracking: a case study. BEHAVIOUR & INFORMATION TECHNOLOGY. 2018

Article information
Achananuparp, P; Lim, EP; Abhishek, V. Does Journaling Encourage Healthier Choices? Analyzing Healthy Eating Behaviors of Food Journalers. DH '18: PROCEEDINGS OF THE 2018 INTERNATIONAL CONFERENCE ON DIGITAL HEALTH. 2018
Skirpan, M; Cameron, J; Yeh, T. Quantified Self: An Interdisciplinary Immersive Theater Project Supporting a Collaborative Learning Environment for CS Ethics. SIGCSE'18: PROCEEDINGS OF THE 49TH ACM TECHNICAL SYMPOSIUM ON COMPUTER SCIENCE EDUCATION. 2018
Wilmott, C; Fraser, E; Lammes, S. I am he. I am he. Siri rules': Work and play with the Apple Watch. EUROPEAN JOURNAL OF CULTURAL STUDIES. 2018
Gerhard, U; Hepp, A. Appropriating Digital Traces of Self-Quantification: Contextualizing Pragmatic and Enthusiast Self-Trackers. INTERNATIONAL JOURNAL OF COMMUNICATION. 2018
Heikkilä, P; Honka, A; Mach, S; Schmalfuss, F; Kaasinen, E; Väänänen, K. Quantified Factory Worker - Expert Evaluation and Ethical Considerations of Wearable Self-tracking Devices. MINDTREK'18: PROCEEDINGS OF THE 22ND INTERNATIONAL ACADEMIC MINDTREK CONFERENCE. 2018
Skirpan, MW; Cameron, J; Yeh, T. More Than a Show: Using Personalized Immersive Theater to Educate and Engage the Public in Technology Ethics. PROCEEDINGS OF THE 2018 CHI CONFERENCE ON HUMAN FACTORS IN COMPUTING SYSTEMS (CHI 2018). 2018
Ben Allouch, S; van Velsen, L. Fit by Bits: An Explorative Study of Sports Physiotherapists' Perception of Quantified Self Technologies. BUILDING CONTINENTS OF KNOWLEDGE IN OCEANS OF DATA: THE FUTURE OF CO-CREATED EHEALTH. 2018
Didziokaite, G; Saukko, P; Greiffenhagen, C. Doing Calories: The Practices of Dieting Using Calorie Counting App MyFitnessPal. METRIC CULTURE: ONTOLOGIES OF SELF-TRACKING PRACTICES. 2018
Katz, DS; Price, BA; Holland, S; Dalton, NS. Designing for Diabetes Decision Support Systems with Fluid Contextual Reasoning. PROCEEDINGS OF THE 2018 CHI CONFERENCE ON HUMAN FACTORS IN COMPUTING SYSTEMS (CHI 2018). 2018
Achananuparp, P; Lim, EP; Abhishek, V; Yun, TJ. Eat & Tell: A Randomized Trial of Random-Loss Incentive to Increase Dietary Self-Tracking Compliance. DH '18: PROCEEDINGS OF THE 2018 INTERNATIONAL CONFERENCE ON DIGITAL HEALTH. 2018
Igo, SE. Me and My Data. HISTORICAL STUDIES IN THE NATURAL SCIENCES. 2018
Akyaman, S; Önol, TA. A CONTENT ANALYSIS OF WIRED MAGAZINE IN TERMS OF SELF-TRACKING DEVICES. TURKISH ONLINE JOURNAL OF DESIGN ART AND COMMUNICATION. 2018
Faust, L; Hachen, D; Lizardo, O; Chawla, NV. Quantifying Subjective Well-being Using Trends in Weekend Activity. 2018 IEEE INTERNATIONAL CONFERENCE ON HEALTHCARE INFORMATICS (ICHI). 2018
Christiansen, TB; Kristensen, DB; Larsen, JE. The 1-Person Laboratory of the Quantified Self Community. METRIC CULTURE: ONTOLOGIES OF SELF-TRACKING PRACTICES. 2018
Staples, WG. Real-time Grade Books and the Quantified Student. METRIC CULTURE: ONTOLOGIES OF SELF-TRACKING PRACTICES. 2018
Kooiman TJM, de Groot M, Hoogenberg K, Krijnen WP, van der Schans CP, Kooy A.. Self-tracking of Physical Activity in People With Type 2 Diabetes: A Randomized Controlled Trial. Comput Inform Nurs. 2018 Jul. 2018
Hassan, L; Dias, A; Hamari, J. How motivational feedback increases user's benefits and continued use: A study on gamification, quantified-self and social networking. INTERNATIONAL JOURNAL OF INFORMATION MANAGEMENT. 2019
Attig, C; Franke, T. I track, therefore I walk - Exploring the motivational costs of wearing activity trackers in actual users. INTERNATIONAL JOURNAL OF HUMAN-COMPUTER STUDIES. 2019

**Article information**
Reychav, I; Beeri, R; Balapour, A; Raban, DR; Sabherwal, R; Azuri, J. How reliable are self-assessments using mobile technology in healthcare? The effects of technology identity and self-efficacy. COMPUTERS IN HUMAN BEHAVIOR. 2019
Stiglbauer, B; Weber, S; Batinic, B. Does your health really benefit from using a self-tracking device? Evidence from a longitudinal randomized control trial. COMPUTERS IN HUMAN BEHAVIOR. 2019
Müller, AM; Wang, NX; Yao, JL; Tan, CS; Low, ICC; Lim, N; Tan, J; Tan, A; Müller-Riemenschneider, F. Heart Rate Measures From Wrist-Worn Activity Trackers in a Laboratory and Free-Living Setting: Validation Study. JMIR MHEALTH AND UHEALTH. 2019
Pols, J; Willems, D; Aanestad, M. Making sense with numbers. Unravelling ethico-psychological subjects in practices of self-quantification. SOCIOLOGY OF HEALTH & ILLNESS. 2019
Bove, LA. Increasing Patient Engagement Through the Use of Wearable Technology. JNP- THE JOURNAL FOR NURSE PRACTITIONERS. 2019
Thévenot, L. Measure for Measure: Politics of Quantifying Individuals to Govern Them. HISTORICAL SOCIAL RESEARCH-HISTORISCHE SOZIALFORSCHUNG. 2019
Wallenburg, I; Bal, R. The gaming healthcare practitioner: How practices of datafication and gamification reconfigure care. HEALTH INFORMATICS JOURNAL. 2019
Sagl, G; Resch, B; Petutschnig, A; Kyriakou, K; Liedlgruber, M; Wilhelm, FH. Wearables and the Quantified Self: Systematic Benchmarking of Physiological Sensors. SENSORS. 2019
Gabriels, K; Coeckelbergh, M. Technologies of the self and other': how self-tracking technologies also shape the other. JOURNAL OF INFORMATION COMMUNICATION & ETHICS IN SOCIETY. 2019
Piras, EM. Beyond self-tracking: Exploring and unpacking four emerging labels of patient data work. HEALTH INFORMATICS JOURNAL. 2019
Lee, VR. On researching activity tracking to support learning: a retrospective. INFORMATION AND LEARNING SCIENCES. 2019
Khakurel, J; Immonen, M; Porras, J; Knutas, A. Understanding the Adoption of Quantified Self-Tracking Wearable Devices in the Organization Environment: An Empirical Case Study. 12TH ACM INTERNATIONAL CONFERENCE ON PERVASIVE TECHNOLOGIES RELATED TO ASSISTIVE ENVIRONMENTS (PETRA 2019). 2019
Kou, YB; Gui, XN; Chen, YA; Nardi, B. Turn to the Self in Human-Computer Interaction: Care of the Self in Negotiating the Human-Technology Relationship. CHI 2019: PROCEEDINGS OF THE 2019 CHI CONFERENCE ON HUMAN FACTORS IN COMPUTING SYSTEMS. 2019
Hepworth, K. A Panopticon on My Wrist: The Biopower of Big Data Visualization for Wearables. DESIGN AND CULTURE. 2019
McKinney, P; Martin, A; Sbaffi, L. Information Literacy in Food and Activity Tracking Among Parkrunners, People With Type 2 Diabetes, and People With Irritable Bowel Syndrome: Exploratory Study. JOURNAL OF MEDICAL INTERNET RESEARCH. 2019
Attig, C; Karp, A; Franke, T. User Diversity in the Motivation for Wearable Activity Tracking: A Predictor for Usage Intensity?. PROCEEDINGS OF THE 20TH CONGRESS OF THE INTERNATIONAL ERGONOMICS ASSOCIATION (IEA 2018), VOL V: HUMAN SIMULATION AND VIRTUAL ENVIRONMENTS, WORK WITH COMPUTING SYSTEMS (WWCS), PROCESS CONTROL. 2019
Nadarajah, SG; Pedersen, PW; Hougaard, BI; Knoche, H. Am I Coughing More Than Usual? Patient Reflections and User Needs on Tracking COPD Data in a Telehealth System. PROCEEDINGS OF THE 4TH INTERNATIONAL WORKSHOP ON MULTIMEDIA FOR PERSONAL HEALTH & HEALTH CARE (HEALTHMEDIA'19). 2019
White, G; Liang, ZL; Clarke, S. A Quantified-Self Framework for Exploring and Enhancing Personal Productivity. 2019 INTERNATIONAL CONFERENCE ON CONTENT-BASED MULTIMEDIA INDEXING (CBMI). 2019

Article information
Leslie, E. This other atmosphere: against human resources, emoji and devices. JOURNAL OF VISUAL CULTURE. 2019
Huppert, F; Kranz, M; Hoelzl, G. Would I Lie to You - Would You Notice?. MOBILE COMPUTING, APPLICATIONS, AND SERVICES, MOBICASE 2019. 2019
Grant AD, Wolf GI, Nebeker C.. Approaches to governance of participant-led research: a qualitative case study. BMJ Open. 2019 Apr 2. 2019
Grant AD, Wolf GI.. Free-Living Humans Cross Cardiovascular Disease Risk Categories Due to Daily Rhythms in Cholesterol and Triglycerides. J Circadian Rhythms. 2019 Apr 24. 2019
Isin, E; Ruppert, E. The birth of sensory power: How a pandemic made it visible?. BIG DATA & SOCIETY. 2020
Esmonde, K. There's only so much data you can handle in your life': accommodating and resisting self-surveillance in women's running and fitness tracking practices. QUALITATIVE RESEARCH IN SPORT EXERCISE AND HEALTH. 2020
Giannakos, MN; Sharma, K; Papavlasopoulou, S; Pappas, IO; Kostakos, V. Fitbit for learning: Towards capturing the learning experience using wearable sensing. INTERNATIONAL JOURNAL OF HUMAN-COMPUTER STUDIES. 2020
De Moya, JF; Pallud, J. From panopticon to heautopticon: A new form of surveillance introduced by quantified-self practices. INFORMATION SYSTEMS JOURNAL. 2020
Leszczynski, A. Digital methods III: The digital mundane. PROGRESS IN HUMAN GEOGRAPHY. 2020
Heyen, NB. From self-tracking to self-expertise: The production of self-related knowledge by doing personal science. PUBLIC UNDERSTANDING OF SCIENCE. 2020
Esmonde, K; Jette, S. Assembling the 'Fitbit subject': A Foucauldian-sociomaterialist examination of social class, gender and self-surveillance on Fitbit community message boards. HEALTH. 2020
DuFault, BL; Schouten, JW. Self-quantification and the datapreneurial consumer identity. CONSUMPTION MARKETS & CULTURE. 2020
Abtahi, P; Ding, V; Yang, AC; Bruzzese, T; Romanos, AB; Murnane, EL; Follmer, S; Landay, JA. Understanding Physical Practices and the Role of Technology in Manual Self-Tracking. PROCEEDINGS OF THE ACM ON INTERACTIVE MOBILE WEARABLE AND UBIQUITOUS TECHNOLOGIES-IMWUT. 2020
Hepp, A. The fragility of curating a pioneer community: Deep mediatization and the spread of the Quantified Self and Maker movements. INTERNATIONAL JOURNAL OF CULTURAL STUDIES. 2020
Wittkowski, K; Klein, JF; Falk, T; Schepers, JJL; Aspara, J; Bergner, KN. What Gets Measured Gets Done: Can Self-Tracking Technologies Enhance Advice Compliance?. JOURNAL OF SERVICE RESEARCH. 2020
Jung, JS; Wellard-Cole, L; Cai, C; Koprinska, I; Yacef, K; Allman-Farinelli, M; Kay, J. Foundations for Systematic Evaluation and Benchmarking of a Mobile Food Logger in a Large-scale Nutrition Study. PROCEEDINGS OF THE ACM ON INTERACTIVE MOBILE WEARABLE AND UBIQUITOUS TECHNOLOGIES-IMWUT. 2020
Ajana, B. Personal metrics: Users' experiences and perceptions of self-tracking practices and data. SOCIAL SCIENCE INFORMATION SUR LES SCIENCES SOCIALES. 2020
Ensari, I; Pichon, A; Lipsky-Gorman, S; Bakken, S; Elhadad, N. Augmenting the Clinical Data Sources for Enigmatic Diseases: A Cross-Sectional Study of Self-Tracking Data and Clinical Documentation in Endometriosis. APPLIED CLINICAL INFORMATICS. 2020
Wienroth, M; Thomsen, LLH; Hostgaard, AM. Health technology identities and self. Patients' appropriation of an assistive device for self-management of chronic illness. SOCIOLOGY OF HEALTH & ILLNESS. 2020
Liang, ZL; Ploderer, B. How Does Fitbit Measure Brainwaves: A Qualitative Study into the Credibility of Sleep-tracking Technologies. PROCEEDINGS OF THE ACM ON INTERACTIVE MOBILE WEARABLE AND UBIQUITOUS TECHNOLOGIES-IMWUT. 2020

**Article information**
Brinson, NH; Rutherford, DN. Privacy and the quantified self: A review of US health information policy limitations related to wearable technologies. JOURNAL OF CONSUMER AFFAIRS. 2020
Hanci, E; Lacroix, J; Ruijten, PAM; Haans, A; IJsselsteijn, W. Measuring commitment to self-tracking: development of the C2ST scale. PERSONAL AND UBIQUITOUS COMPUTING. 2020
Quaglia, V. Men, masculinities and diabetes: 'doing gender' in Italian men's narratives of chronic illness. SOCIOLOGY OF HEALTH & ILLNESS. 2020
Yang, N; van Hout, G; Feijs, L; Chen, W; Hu, J. Facilitating Physical Activity through On-Site Quantified-Self Data Sharing. SUSTAINABILITY. 2020
Haluza, D; Böhm, I. The Quantified Woman: Exploring Perceptions on Health App Use among Austrian Females of Reproductive Age. REPRODUCTIVE MEDICINE. 2020
Maturo, A; Shea, M. THE QUANTIFIED SELF OR THE MARKETIZED SELF? HOW DATA AND THE DRIVE TO OPTIMIZE LEAD TO NEOLIBERAL PERFORMANCE CULTURE. BALKAN JOURNAL OF PHILOSOPHY. 2020
Liu, Y; Lv, XY; Tang, ZY. The impact of mortality salience on quantified self behavior during the COVID-19 pandemic. PERSONALITY AND INDIVIDUAL DIFFERENCES. 2021
Eikey, EV. Effects of diet and fitness apps on eating disorder behaviours: qualitative study. BJPSYCH OPEN. 2021
Hepp, A; Alpen, S; Simon, P. Beyond empowerment, experimentation and reasoning: The public discourse around the Quantified Self movement. COMMUNICATIONS-EUROPEAN JOURNAL OF COMMUNICATION RESEARCH. 2021
Heikkilä, P; Honka, A; Kaasinen, E; Väänänen, K. Quantified factory worker: field study of a web application supporting work well-being and productivity. COGNITION TECHNOLOGY & WORK. 2021
Robards, B; Lyall, B; Moran, C. Confessional data selfies and intimate digital traces. NEW MEDIA & SOCIETY. 2021
Tan, SHA; Smith, TEL. An optimal environment for our optimal selves? An autoethnographic account of self-tracking personal exposure to air pollution. AREA. 2021
Tzovaras, BG; Hidalgo, ES; Alexiou, K; Baldy, L; Morane, B; Bussod, I; Fribourg, M; Wac, K; Wolf, G; Ball, M. Using an Individual-Centered Approach to Gain Insights From Wearable Data in the Quantified Flu Platform: Netnography Study. JOURNAL OF MEDICAL INTERNET RESEARCH. 2021
Dolezel, M; Smutny, Z. Usage of eHealth/mHealth Services among Young Czech Adults and the Impact of COVID-19: An Explorative Survey. INTERNATIONAL JOURNAL OF ENVIRONMENTAL RESEARCH AND PUBLIC HEALTH. 2021
Prange, S; Mayer, S; Bittl, ML; Hassib, M; Alt, F. Investigating User Perceptions Towards Wearable Mobile Electromyography. HUMAN-COMPUTER INTERACTION, INTERACT 2021, PT IV. 2021
Butte, KD; Bahmani, A; Butte, AJ; Li, X; Snyder, MP. Five-year pediatric use of a digital wearable fitness device: lessons from a pilot case study. JAMIA OPEN. 2021
Trace, CB; Zhang, Y. Minding the Gap: Creating Meaning from Missing and Anomalous Data. INFORMATION & CULTURE. 2021
Lei, L; Zhu, YL; Liu, Q. Analysis on Quantified Self-Behavior of Customers in Food Consumption under the Perspective of Social Networks. COMPLEXITY. 2021
Hughes, KL. Speaking the Data': Renegotiating the Digitally-Mediated Body Through Performative Embodied Praxis, Sound and Rhythmic Affect. CULTURAL STUDIES-CRITICAL METHODOLOGIES. 2021
Grinberg, Y. The Data Multiple: Seeing Double in Digital Entrepreneurialism. ANTHROPOLOGICAL QUARTERLY. 2021
Rocamora, A. The Datafication and Quantification of Fashion: The Case of Fashion Influencers. FASHION THEORY-THE JOURNAL OF DRESS BODY & CULTURE. 2022

Article information
Jin, H; Peng, Y; Chen, J; Park, ST. Research on the Connotation and Dimension of Consumers' Quantified-Self Consciousness. SUSTAINABILITY. 2022
Sharma, K; Pappas, I; Papavlasopoulou, S; Giannakos, M. Wearable Sensing and Quantified-self to explain Learning Experience. 2022 INTERNATIONAL CONFERENCE ON ADVANCED LEARNING TECHNOLOGIES (ICALT 2022). 2022
Pilgrim, K; Bohnet-Joschko, S. Donating Health Data to Research: Influential Characteristics of Individuals Engaging in Self-Tracking. INTERNATIONAL JOURNAL OF ENVIRONMENTAL RESEARCH AND PUBLIC HEALTH. 2022
Lee, S; Suh, KS. Deliberate news consumption through the quantified self and the self-regulatory process. INFORMATION & MANAGEMENT. 2022
Pilgrim, K; Bohnet-Joschko, S. Effectiveness of Digital Forced-Choice Nudges for Voluntary Data Donation by Health Self-trackers in Germany: Web-Based Experiment. JOURNAL OF MEDICAL INTERNET RESEARCH. 2022
Vermeer, L. From Diaries to Data Doubles. Self-Tracking in Dutch Diaries (1780-1940). LIFE WRITING. 2022
Chiodo, S. Quantified Self as Epistemological Anarchism. PHILOSOPHIA. 2022
Fan, XW; Fan, J; Li, JL. The Effect of Presentation Characteristics of Quantified Self Data on Consumers Continuance Participation Intention: An Empirical Study Based on Health-Related Apps '. PSYCHOLOGY RESEARCH AND BEHAVIOR MANAGEMENT. 2022
Zhao, ZZ; Haikel-Elsabeh, M; Baudier, P; Renard, D; Brem, A. Need for Uniqueness and Word of Mouth in Disruptive Innovation Adoption: The Context of Self-Quantification. IEEE TRANSACTIONS ON ENGINEERING MANAGEMENT. 2023
De Cristofaro, D; Chiodo, S. Quantified Sleep: Self-Tracking Technologies and the Reshaping of 21st-Century Subjectivity. HISTORICAL SOCIAL RESEARCH-HISTORISCHE SOZIALFORSCHUNG. 2023
Gerdenitsch, C; Bieg, T; Gaitsch, M; Schörpf, P; Tscheligi, M; Kriglstein, S. Tracking to Success? A Critical Reflection on Workplace Quantified-Self Technologies from a Humanistic Perspective. PROCEEDINGS OF THE 2ND ANNUAL MEETING OF THE SYMPOSIUM ON HUMAN-COMPUTER INTERACTION FOR WORK, CHIWORK 2023. 2023
Zillien, N; Wettmann, N; Peper, F. Sleep Experiments. Knowledge Production through Self-Tracking. HISTORICAL SOCIAL RESEARCH-HISTORISCHE SOZIALFORSCHUNG. 2023
Hepp, A. The lab, the space and the meetup: locating technological experimentation in everyday life. JCOM-JOURNAL OF SCIENCE COMMUNICATION. 2023
Hoefer, M; Voida, S. Being, Having, Doing, and Interacting: A Personal Informatics Approach to Understanding Human Need Satisfaction in Everyday Life. DESIGNING INTERACTIVE SYSTEMS CONFERENCE, DIS 2023. 2023
Plester, B; Sayers, J; Keen, C. Health and wellness but at what cost? Technology media justifications for wearable technology use in organizations. ORGANIZATION. 2024
Leung, W; Shi, L; Jung, J. Are individuals with disabilities using wearable devices? A secondary data analysis of 2017 BRFSS. DISABILITY AND REHABILITATION-ASSISTIVE TECHNOLOGY. 2024
Esfahani, MJ; Sikder, N; ter Horst, R; Daraie, AH; Appel, K; Weber, FD; Bevelander, KE; Dresler, M. Citizen neuroscience: Wearable technology and open software to study the human brain in its natural habitat. EUROPEAN JOURNAL OF NEUROSCIENCE. 2024
Hepp, A. Curators of digital futures: The life cycle of pioneer communities. NEW MEDIA & SOCIETY. 2024
Wilmont, I; Loeffen, M; Hoogeboom, T. A qualitative study on the facilitators and barriers to adopting the N-of-1 trial methodology as part of clinical practice: potential versus implementation challenges. INTERNATIONAL JOURNAL OF QUALITATIVE STUDIES ON HEALTH AND WELL-BEING. 2024

**Article information**
Li, AX; Ding, ZP; Sun, CH; Liu, YZ. Recommending AI based on Quantified Self: Investigating the mechanism of consumer acceptance of AI recommendations. ELECTRONIC MARKETS. 2024
Asmuss, J; Hepp, A; Lüpkes, J; Schmitz, A. Solving the crisis with do-it-yourself heroes? The media coverage on pioneer communities, Covid-19, and technological solutionism. COMMUNICATIONS-EUROPEAN JOURNAL OF COMMUNICATION RESEARCH. 2024
Rigal, A. On the cult of the individual: The Quantified Self public gatherings, self-tracking, and individualism. CURRENT SOCIOLOGY. 2024
Call, SR; Jensen, JT; Barbour, JB. Quantifying the self with others. BIG DATA & SOCIETY. 2024
Fourie, D. People aren't numbers: A critique of industrial rationality within neoliberal societies. SOUTH AFRICAN JOURNAL OF PHILOSOPHY. 2024
Moravcíková, E; Jakubovská, V; Jakubovská, K. MAN VS. TECHNOLOGY (FORMS AND TRANSFORMATIONS OF VIRTUALISATION OF EVERYDAY LIFE IN THE ERA OF TRANSHUMANISM). COMMUNICATION TODAY. 2024
Liu, JQ; Chen, S. Distal and proximal factors of wearable users' quantified-self dependence: A cognitive-behavioral model. DIGITAL HEALTH. 2024
Sobey, A; Carter, L. The Harmful Fetishisation of Reductive Personal Tracking Metrics in Digital Systems. PROCEEDINGS OF THE 2024 ACM CONFERENCE ON FAIRNESS, ACCOUNTABILITY, AND TRANSPARENCY, ACM FACCT 2024. 2024
Wang, S. Data auxiliaries: making online dating 'work' with dating spreadsheets. INFORMATION COMMUNICATION & SOCIETY. 2025
