# Supplementary material for: Epistemic agency in self-tracking: a conceptual literature review of quantified self as citizen science for health
Source: Front Public Health. 2026 Aug 18;14:1736850. doi: 10.3389/fpubh.2026.1736850 (PMC13530006; doi:10.3389/fpubh.2026.1736850)
Supplement: Supplementary file 2 [file Data_Sheet_2.pdf]

Supplementary table 2. Descriptions of articles included in analysis sorted by articles describing self-trackers (the people) and self-tracking (the activity) for different levels of agency (low/mixed/high) respectively.

Article	Description
ARTICLES WITH LOW LEVELS OF AGENCY	
Describing self-trackers (the people)	
Wang et al. (2016), (1)	A large-scale secondary analysis linking smart-scale data shared via Twitter with users' general tweeting behavior to explore whether social media language and activity patterns could predict body weight. While the data derive from self-tracking practices, the self-trackers themselves did not participate beyond generating automated records. The study exemplifies how passive self-tracking data can be co-opted into academic research, generating predictive models without participant agency or reflection. It illustrates the epistemic gap between self-tracking as a personal practice and self-tracking as a data source for conventional research.
Smith and Vonthethoff (2017), (2)	Analyzes how members of the QS community engage with self-tracking to understand their bodies through data. The study argues that datafication creates a new form of "unbodied" embodiment where individuals outsource self-knowledge to algorithmic systems. While self-trackers seek self-knowledge and agency, their reasoning often relies on the authority of data, leading to a partial displacement of bodily intuition. The researchers conclude that self-tracking simultaneously empowers and disciplines individuals, turning self-care into a technologically mediated moral project rather than a fully self-determined scientific inquiry.
Gerhard and Hepp (2018), (3)	Investigates everyday self-tracking through a media-ethnographic study, distinguishing between pragmatic users who track instrumentally and enthusiasts who integrate tracking into lifestyle and identity. Even when self-tracking appears autonomous, it reproduces collective norms and technological authority. Thus, while the self-tracking described generates personal data, it does not reflect a personal science process, that is, the individuals participate in data production but not in defining questions, analyzing results, or articulating meaning from their own lived experience.
Describing self-tracking (the activity)	
Krijnen et al. (2013), (4)	A project at a university where students and staff used QS tools—specifically Fitbit Zip pedometers and GPS trackers—to visualize and reflect on their daily activities. The overall aim of the project, formulated by the researchers, was to explore whether data from these devices could be used to map daily habits and raise awareness of sedentary behavior. Participants were thus not involved in developing the goals or research questions of the study. Their role consisted primarily of collecting their own step counts and location data over a two-month period, following the instructions provided by the research team. The subsequent data analysis, including the visualization of step patterns, gender differences, and movement maps, was conducted entirely by the researchers. Likewise, the interpretation of findings and articulation of lessons learned were handled at the study level rather than by the individual participants.
Meadow et al. (2014), (5)	Study of phone microbiomes. Workshop participants swabbed their own phone screens and fingers to compare bacterial communities. All analysis (sequencing, statistics) was done by authors.
Mayor & Gamaiunova (2014), (6)	Mood/distraction and stress with wearable where 66 adults wore a wrist HR monitor during stress tasks. All study design, data analysis and interpretation were done by researchers.
Walker Rettberg (2014), (7)	Analyzes the cultural and personal dimensions of the QS movement, exploring how wearable sensors, apps, and digital platforms turn everyday life into measurable data. She argues that self-tracking extends traditional forms of self-representation—like diaries or photographs—into the digital realm, producing data-driven "images" of the self that feel objective yet remain deeply interpretive.

	Through examples ranging from fitness trackers and baby monitors to sleep and sex apps, Rettberg demonstrates how dataism—the belief that everything valuable can be quantified—shapes modern subjectivity. Rettberg situates this within broader cultural systems of control and self-optimization, showing that while self-tracking promises self-knowledge, it often narrows experience to what technology can measure, displacing reflective agency with quantified representation.
Gollamudi et al. (2016), (8)	Hypertension trial analysis where 38 patients recorded 6,290 BP readings via smartphone home-monitoring. The researchers defined the goals and used statistical models to analyze the time-series data. The participants did not analyze or draw conclusions from their own data.
Verdezoto & Grønvall (2016), (9)	Workshop study of home BP self-monitoring in older adults. Participants were given digital BP cuffs and measured and recorded their own BP at home (Observing). In follow-up sessions they shared their measurements, reflected on issues, and gave feedback on sample data visualizations, however not sufficient to correspond to Reasoning or Discovering. Questioning by researchers.
Rapp & Cena (2016), (10)	Diary study of 14 novices using trackers. Participants were given wearable/smartphone apps to record activity, sleep, diet and mood data. They collected data (Observing) and kept diaries reflecting on use, but the research question was set by authors. The study notes participants "considered the act of collecting personal information burdensome", showing their own reflections, however, this is not sufficient to count as Discovering. Data analysis and interpretation were done by the researchers (Questioning and Reasoning by researcher only).
Clinch et al. (2016), (11)	Experimental lifelogging study in which researchers equipped themselves with wearable cameras and sensors to document shared experiences in a controlled, instrumented house. The project aimed to identify methodological and ethical challenges in collecting high-density personal data. The participants' engagement in data capture served institutional research goals rather than individual reflection or learning. The article thus exemplifies a meta-self-tracking exercise: researchers studying self-tracking practices without engaging in the full cycle of personal science. It contributes to the technical and ethical understanding of lifelogging but not to self-generated or experiential knowledge creation by the individuals involved.
Hermsen et al. (2017), (12)	711 adults received Fitbit Zips and recorded on average 129 days of activity over 10 months. Participants wore the devices and generated step data. Researchers formulated the study and analyzed the decline in usage.
Faust et al. (2018), (13)	This study had 125 college students wear Fitbits for two years to record step counts. Researchers identified distinct patterns (high vs. low weekend activity) by clustering the data, then linked these patterns to well-being measures. The research question and analysis were formulated and conducted by the investigators; the student subjects only collected the data by wearing the devices.
Chrisinger & King (2018), (14)	Built-environment pilot integrating a stress sensor. Participants wore a biometric wrist sensor and used a smartphone app (Discovery Tool) to collect photos/audio about neighborhood features. The researchers provided the app and study goals. After data collection, interactive maps of each participant's stress data were shared with them and participants verbally identified neighborhood features affecting well-being. Final analysis for the paper was by researchers.
Kooiman et al. (2019), (15)	RCT of Fitbit in Type-2 diabetes where 72 patients were randomized to a web+tracker program. Those in the intervention wore Fitbits and logged steps daily. The research questions (effect on activity/HbA1c) and analysis were by investigators. Patients did not perform their own data analysis – they simply used the device. The paper notes 90% of participants found the tracker useful, but study design and interpretation were by researchers.
Huppert et al. (2019), (16)	Investigates how people perceive and trust data accuracy from fitness trackers when presented with manipulated feedback. The authors—not the participants—formulated the research question and designed a controlled field experiment to explore whether altered pedometer readings affect perceived credibility. Participants wore devices during their daily walks, collecting step-count data according

	to instructions provided by the researchers, but they neither defined what to track nor analyzed their data. The analysis, visualization, and interpretation of results were entirely handled by the researchers, who found that users continued to trust their devices even when data were intentionally falsified. The participants themselves did not articulate personal reflections or draw conclusions.
Ensari et al. (2020), (17)	Endometriosis tracking vs. EHR. The study analyzed self-reported symptom data from 6,925 app users. Participants used a symptom-tracking app, but all analysis (calculating prevalence, variability) was done by the authors. The participants did not define hypotheses or analyze the aggregate data.
ARTICLES WITH MIXED LEVELS OF AGENCY	
Describing self-trackers (the people)	
Ruckenstein (2014), (18)	Investigates how individuals engage with personal analytics through the concept of the data double—digital representations of the self generated by tracking technologies. Participants in a Finnish study used heart-rate variability monitors to explore stress and recovery but within a researcher-defined project. In the Observing phase, participants contributed physiological data and diaries; they participated in collective Reasoning by interpreting visualized reports in feedback sessions. While the study highlights participants' affective engagement with their data, the inquiry's goals and interpretive structure were externally determined. Thus, self-tracking here functions mainly as a research tool rather than a personal investigation—an instance of conventional research incorporating participatory reflection, rather than personal science.<br><br>Questioning and Discovering are entirely researcher-driven, Observing and Reasoning contain elements of agency on the part of the individual self-tracker.
Baron et al. (2017), (19)	Describes patients whose dependence on sleep-tracking devices created a feedback loop of anxiety and misperception—termed orthosomnia. The individuals' self-tracking lacked reflective purpose and autonomy: they interpreted device-generated data as objective truth, dismissing professional assessments. The study underscores how self-tracking can foster compulsive self-surveillance and erode trust in subjective experience. Rather than enabling self-understanding, the practice amplified stress and disrupted sleep, illustrating the risks of low-agency self-tracking where the person becomes a passive data subject governed by algorithmic authority.<br><br>Questioning and Discovering are entirely researcher-driven, Observing and Reasoning contain elements of agency on the part of the individual self-tracker.
Kristensen and Ruckenstein (2018), (20)	Conceptualizes self-tracking as a "laboratory of the self," a dynamic site where individuals and technologies co-evolve through iterative experimentation. Danish self-trackers explore data to improve, understand, or "feel" themselves, yet their engagement fluctuates between agency and fatigue. The study reveals how self-tracking fosters both empowerment and constraint—enhancing sensory awareness and intentionality while often leading to "dead ends" when novelty fades. Ultimately, the article highlights self-tracking as a situated, time-bound mode of inquiry that bridges subjective reflection and data-driven rationality but remains distinct from fully autonomous personal science.<br><br>Although they report from interviews with self-trackers the particular framing of the study means that the process is quite superficial and aspects from the personal science cycle cannot be specifically identified.
Vesnic-Alujevic et al. (2018), (21)	Examines how online communities surrounding Fitbit and QS devices produce and assess lay health knowledge. Users primarily enact the Observing phase—collecting, reporting, and comparing sensor-based data—while Reasoning and Discovering appear in dialogical,

	collective forms. Personal Questioning is minimal; instead, inquiry is distributed across forum discussions. The study frames these interactions as "knowledge assessment," where experiential and auto-ethnographic posts form a kind of post-normal science, blurring boundaries between citizen users and professional experts. While the forums lack methodological rigor, they generate socially embedded, practice-based understanding of health and technology, offering insight into how citizens co-create meanings of self-tracking, health responsibility, and digital care. The authors conclude that wearable sensor communities both reinforce and subtly resist technocratic visions of efficiency, creating new forms of participatory, collective sense-making around personal health.<br><br>Questioning is entirely researcher-driven, Observing, Reasoning, and Discovering contain elements of agency on the part of the individual self-tracker.
Chiodo (2022), (22)	Interprets the QS phenomenon as a manifestation of epistemological anarchism—a condition in which traditional authorities of knowledge (science, medicine, expertise) are replaced by individualized, self-referential systems of knowing. Through analysis of self-tracking cases such as health and happiness monitoring, Chiodo identifies three intertwined epistemic shifts: from feelings to numbers (overreliance on quantification), from expert knowledge to self-expertise (rejection of authority), and from relationality to individualism (isolation of meaning-making). Chiodo concludes that this autonomy paradoxically results in rulerlessness: individuals gain freedom from external authorities while surrendering epistemic autonomy to technological automation, revealing a crisis of human knowledge and self-understanding in the algorithmic age.<br><br>Appears to perform all four phases but within an increasingly self-enclosed epistemic system, Questioning stems from a desire for control and certainty, Observing centers on quantifying the body, Reasoning collapses into algorithmic or self-referential logic, Discovering becomes personal justification rather than shared understanding.
De Christofaro and Chiodo's (2023), (23)	An analysis of sleep-tracking can be interpreted through the personal science framework as an incomplete and externally driven cycle. The Questioning phase—how to sleep better—is present but shaped by social and commercial discourses rather than personal curiosity or autonomous inquiry. In Observing, individuals collect detailed sleep data through wearables, yet the parameters and interpretations are predefined by the technologies. Reasoning is largely absent at the personal level: users rarely analyze or experiment with their own data but instead defer to the device's algorithms as epistemic authorities. Similarly, Discovering—the reflective articulation of insights—is outsourced to the technology's feedback systems or to broader cultural narratives about health and productivity. Thus, sleep-tracking, as portrayed by De Cristofaro and Chiodo, illustrates a hollow form of personal science: a cycle dominated by Observation without genuine self-directed Questioning, Reasoning, or Discovering—producing data-rich but knowledge-poor self-understanding.<br><br>Reasoning is entirely researcher-driven, Questioning, Observing and Discovering contain elements of agency on the part of the individual self-tracker.
Call et al. (2024), (24)	Explores how self-tracking can become a collaborative practice through their ethnographic study of an online community. In weekly meetings, members present personal tracking projects, share findings, and discuss issues of data interpretation, ethics, and openness. The study highlights how personal science can be both individual and communal—data collection and interpretation are deeply personal, yet understanding is co-produced through dialogue and critique. This collective approach redefines autonomy not as isolation

	but as shared agency within networks of mutual learning. The authors argue that such communities embody participatory, ethical, and democratic models of self-knowledge and digital health innovation.<br><br>Presents different examples, in one case agency is high, in the other low
Describing self-tracking (the activity)	
Whitson (2013), (25)	Reflects on her own experiences with gamified self-tracking tools to explore how pleasure and play sustain voluntary surveillance. Through Observing, she examines her daily engagement with digital platforms that quantify productivity and well-being. Her Reasoning connects these experiences to surveillance theory, arguing that gamification transforms Foucault's "care of the self" into algorithmic self-management. Finally, in Discovering, Whitson concludes that gamified self-tracking seduces individuals into self-regulation by making surveillance enjoyable. The work combines self-observation with critical analysis, offering an example of reflective self-tracking as both empirical material and conceptual lens—an instance of researcher-as-participant inquiry that approaches but does not fully enact personal science.<br><br>Demonstrates agency in Observing and to some degree in Reasoning and Discovering but not in Questioning.
Wilmott et al. (2018), (26)	This article investigates everyday interactions with the Apple Watch as a site where play, labor, and surveillance intertwine. Through ethnographic and phenomenological analysis, the authors explore how wearable technology mediates users' sense of self, productivity, and bodily awareness. Applying the personal science framework, the study reflects on how users engage in self-tracking but primarily within corporate design constraints rather than through self-defined inquiry.<br><br>Demonstrates agency in Observing and to some degree in Reasoning and Discovering but not in Questioning.
Hepworth (2019), (27)	Examines the author's own experience of self-tracking with a Fitbit to reveal how data visualization mediates power and self-governance in contemporary digital culture. Guided by Foucauldian concepts of biopower and the Panopticon, Hepworth explores how the visual aesthetics and gamified features of Fitbit's interface—such as comparisons, leaderboards, and bright, "friendly" graphics—encourage users to monitor and optimize themselves in accordance with neoliberal ideals of self-management. Through reflective engagement with her own data on heart rate, sleep, and activity, she argues that self-tracking can seduce through feelings of control and empowerment while simultaneously subjecting users to surveillance and corporate data extraction, and that personal data visualizations function as instruments of governance, shaping individuals' sense of self and agency. She concludes that ethical design practices and interdisciplinary collaboration are essential to counteract the manipulative and potentially harmful effects of wearable technologies.<br><br>Demonstrates agency in Observing and to some degree in Reasoning and Discovering but not in Questioning.
ARTICLES WITH HIGH LEVELS OF AGENCY	
Describing self-trackers (the people)	
Swan (2013), (28)	QS is framed as both a personal science and a disruptive paradigm for big data research. Self-trackers pursue individual questions of wellbeing, performance, and health optimization, collecting diverse data streams and reasoning through iterative analysis. The movement's epistemology is based on subjective meaning-making, reframing science as participatory and iterative. Swan envisions self-tracking as an engine for discovery that extends from individual insight to collective intelligence, transforming personal data into a

	new infrastructure for biological and behavioral understanding. The article positions self-trackers as high-agency knowledge producers—scientists of their own bodies contributing to new data-driven research methods.
Choe et al. (2014), (29)	Examines how QS practitioners engage in self-tracking as a form of informal personal science. The study highlights both empowerment and limits: QS fosters creativity, self-reflection, and health insight, yet struggles with over-tracking, missing context, and weak analytic frameworks. By capturing the lived practices of "extreme users," the authors reveal how self-tracking operates as an emergent grassroots form of scientific inquiry—motivated by curiosity and lived relevance rather than conventional academic structure.
Nafus and Sherman (2014), (30)	Depicts the QS movement as a site of soft resistance to the biopolitics of big data, where individuals use self-tracking to reclaim interpretive authority over personal information. This ethnography reframes QS as a space of mindful experimentation and autonomy, where data becomes a medium of reflection rather than surveillance—offering an alternative vision of analytics grounded in personal meaning, rather than institutional control.
Sharon and Zandbergen (2017), (31)	Reframes self-tracking as a reflective and relational process rather than a form of data fetishism. Self-tracking thus serves as a practice of mindfulness, a means of resistance to normative systems, and a communicative act bridging private experience and shared understanding. Rather than treating data as objective truth, participants use it to augment awareness, reclaim agency, and narrate their lives. The article positions QS not as naive data worship but as an emergent form of quantifying selfhood—a way of producing knowledge that is embodied, situated, and socially meaningful.
Dolejšová & Kera (2017), (32)	Presents soylent self-experimentation as an example of extreme citizen science, where individuals design, test, and share dietary experiments outside institutional frameworks. The study—based on 11 months of ethnographic fieldwork—shows that these practices enhanced participants' nutrition literacy and sense of agency but also revealed vulnerabilities: collective rationalization, limited methodological rigor, weak accountability, and privacy risks. The authors conclude that while soylent self-experimenters embodied citizen-driven scientific engagement, they require clearer protocols and ethical guidance to transform community curiosity into credible, safe, and sustainable self-research.
Heyen (2019), (33)	Offers a systematic sociological analysis of how self-trackers transform their own data into verified, practical self-knowledge. Through self-posed questions, systematic observation, reflective reasoning, and self-applied explanations, individuals generate *verified and practical self-knowledge*—a form of *self-expertise*. Drawing on interviews and ethnographic observation, Heyen shows how self-trackers use scientific methods (hypothesis testing, data analysis, quality control) to answer personally relevant questions. While these insights may seem trivial to professional science, they have deep practical meaning for the individual and challenge conventional boundaries between science and society. Personal science thus represents a new, self-directed mode of public engagement with science—one that individualizes knowledge production and situates the self as both subject and scientist.
Robards et al. (2021), (34)	Conceptualizes "confessional data selfies" as a new form of digital self-expression blending self-tracking with storytelling. On Reddit's r/DataIsBeautiful, users transform personal data into visually compelling narratives about relationships, grief, or mental health. They use self-tracking to seek meaning, connection, and validation. Their data become emotional, aesthetic, and relational—an act of confession and self-understanding rather than control. The article reframes self-tracking from a tool of surveillance or productivity toward a creative, affective, and socially mediated practice of making sense of life through data.
Vermeer (2022), (35)	Examines early forms of self-tracking through five historical diaries, showing that the urge to monitor one's body and habits long predates digital wearables. Vermeer argues that these diaries demonstrate an early intertwining of the quantified and qualified self, where self-tracking was both numerical and interpretive. The article situates this practice within emerging norms of "ableism" and self-governance, showing strong continuity between historical diaries and modern digital self-tracking as "technologies of the self."

Zillien et al. (2023), (36)	Conceptualizes sleep self-tracking as an experimental, knowledge-producing practice among “expertized laypersons.” Drawing on ethnographic analysis of blogs and videos from QS and biohacking communities, they show how individuals use sleep-tracking devices to explore personal questions such as “How can I improve my sleep?” These self-experiments blend objective data with subjective reflection, creating hybrid forms of lay expertise. The authors argue that such practices fill gaps left by medical uncertainty, turning self-tracking into a form of everyday science. Rather than replacing professional knowledge, sleep self-experimentation complements it by producing experiential, situated understanding—demonstrating how personal science can generate credible, practice-based knowledge outside traditional institutions.
Rigal (2024), (37)	Investigates how the QS movement embodies what Émile Durkheim called the “cult of the individual,” a form of collective ritual centered on the value of personal autonomy. Drawing on Randall Collins’ interaction ritual theory, Rigal analyzes 369 videos of QS “show-and-tell” presentations to identify how self-focused practices become collective. He shows that QS events feature ritual ingredients—co-presence, shared focus on personal data, rhythmic presentation structures, and symbolic protection of individual autonomy—creating a transnational “interaction ritual chain” across 71 cities. These gatherings celebrate singular, data-based selfhood while sustaining community cohesion.
Describing self-tracking (the activity)	
Magnuson et al. (2016), (38)	Presents a rigorously designed N-of-1 self-experiment examining the effects of antidepressant and sleep-aid polypharmacy on sleep quality. She articulated how individualized N-of-1 protocols can guide personalized treatment decisions, thereby generating new questions about safe deprescribing in psychiatric contexts.
Riggare et al. (2017), (39)	Presents a self-initiated, placebo-controlled N-of-1 trial exploring nicotine’s potential to reduce dyskinesia in Parkinson’s disease. The study not only demonstrated a perceived therapeutic effect of nicotine but also articulated the epistemic and ethical dimensions of patient-driven research. By framing self-tracking as legitimate scientific inquiry, it illustrated how patients can generate individualized, actionable knowledge while contributing to broader methodological discourse. The article thus exemplifies personal science as both a practical and epistemological innovation—where the patient acts simultaneously as subject, researcher, and communicator of new medical understanding.
Lee et al. (2017), (40)	A collaborative N-of-1 case study comparing manually logged and CGM-based blood glucose data from a child with Type 1 diabetes. The analysis revealed how habitual schedules and technological interfaces shape what data are noticed or overlooked. Although CGM provided more continuous data, the family rarely used aggregated visualizations until prompted by a physician—highlighting how access alone does not guarantee reflection. The paper illustrates personal science as lived collaboration between patients and researchers, showing how systematic self-tracking and reflective reasoning can generate actionable self-knowledge and inform design and clinical practices alike.
Marcengo et al. (2017), (41)	This 11-month autoethnographic study investigates how a mindfulness practitioner used an EEG headband to explore attention, distraction, and the role of neurofeedback in sustaining meditation practice. Overall, the work exemplifies *personal science*—iterative self-inquiry blending experiential reflection and empirical measurement to refine mindful awareness.
Christiansen et al. (2018), (42)	Introduces the concept of the 1-Person Laboratory (1PL) to describe how members of the Quantified Self community transform everyday life into sites of scientific inquiry. Through autoethnographic examples and community analysis, they show that self-trackers act as both subjects and researchers, using systematic experimentation to address personally meaningful questions about health, productivity, and well-being. The authors argue that the 1PL blurs boundaries between science and life, producing situated, experiential knowledge that complements traditional research. Rather than challenging science, personal science democratizes it—making

	experimentation accessible, reflexive, and personally relevant. The 1PL thus exemplifies autonomy and creativity in everyday knowledge production.
Grant and Wolf (2019), (43)	Demonstrates how self-trackers can collectively advance biomedical insight through participant-led research. Twenty-one individuals designed and conducted personal experiments on lipid variability, using at-home assays to test hypotheses about daily, ultradian, and menstrual rhythms. Results showed that all participants crossed cardiovascular risk categories within a day, revealing substantial temporal dynamics in cholesterol and triglycerides. The study exemplifies how distributed self-tracking can yield both individual learning and generalizable knowledge—bridging the gap between citizen curiosity and clinical science. It redefines the self-tracker as an autonomous yet collaborative investigator, capable of producing clinically relevant insights under everyday conditions.
White et al. (2019), (44)	Proposes a framework based on a one-year single-subject study using self-tracking to explore how daily behaviors influence academic productivity. This study exemplifies genuine personal science: self-directed, data-driven inquiry producing both individual insight and methodological contribution. It demonstrates how structured self-tracking can transform everyday behaviors into systematic experimentation, bridging personal learning and scientific rigor.
Tan and Smith (2021), (45)	A personal, reflexive exploration of air-pollution self-tracking through an environmental sensor. The findings reveal that self-tracking cultivates curiosity and dialogue rather than direct behavioural transformation. While data visualizations provoke awareness, real-world constraints—mobility, geography, culture—limit change. The article reframes self-tracking as an ecological and social practice: rather than striving for the "optimal self," it envisions the "optimal environment for our optimal selves," where personal sensing contributes to shared understanding and civic engagement around environmental health.
Butte et al. (2021), (46)	Describes a five-year n-of-1 case study of a teenage girl and her father who continuously tracked their steps and sleep using Fitbit devices. Initially motivated by personal curiosity about activity patterns and health, their self-tracking evolved into a formal study examining long-term wearable use in a pediatric context, making this a model of hybrid personal and academic inquiry that demonstrates how long-term self-tracking can yield both personal insight and broader scientific understanding.

1. Wang Y, Weber I, Mitra P. Quantified Self Meets Social Media: Sharing of Weight Updates on Twitter. In: Proceedings of the 6th International Conference on Digital Health Conference [Internet]. Montréal Québec Canada: ACM; 2016 [cited 2025 Aug 2]. p. 93–7. Available from: https://dl.acm.org/doi/10.1145/2896338.2896363

2. Smith GJD, Vonthethoff B. Health by numbers? Exploring the practice and experience of datafied health. Health Sociol Rev. 2017 Jan 2;26(1):6–21.

3. Gerhard U, Hepp A. Appropriating Digital Traces of Self-Quantification: Contextualizing Pragmatic and Enthusiast Self-Trackers. Int J Commun. 2018;12:683–700.

4. Krijnen D, Riphagen M, van Hout M, Gootjes G. Learning Tomorrow: Visualizing Student and Staff Physical Activity. In Barcelona, Spain: International Association of Technology, Education and Development (IATED); 2013.

5. Meadow JF, Altrichter AE, Green JL. Mobile phones carry the personal microbiome of their owners. PeerJ [Internet]. 2014;2014(1). Available from: https://www.scopus.com/inward/record.uri?eid=2-s2.0-84903846612&doi=10.7717%2fpeerj.447&partnerID=40&md5=3740dd6cd3206f0ad94f6b35dd878671

6. Eric M, Gamaiunova L. Using wearable technology for psychophysiological experiments. Gender roles and cognitive appraisal impact cardiac response to socio-evaluative stress. In: Proceedings of the 4th International Conference on Wireless Mobile Communication and Healthcare - “Transforming healthcare through innovations in mobile and wireless technologies” [Internet]. Athens, Greece: ICST; 2014 [cited 2025 Aug 2]. Available from: http://eudl.eu/doi/10.4108/icst.mobihealth.2014.257345

7. Rettberg JW. Quantified Selves. In: Seeing Ourselves Through Technology [Internet]. London: Palgrave Macmillan UK; 2014 [cited 2025 Aug 2]. p. 61–78. Available from: http://link.springer.com/10.1057/9781137476661_5

8. Gollamudi SS, Topol EJ, Wineinger NE. A framework for smartphone-enabled, patient-generated health data analysis. PeerJ [Internet]. 2016;2016(2). Available from: https://www.scopus.com/inward/record.uri?eid=2-s2.0-84992084087&doi=10.7717%2fPEERJ.2284&partnerID=40&md5=4e075c5135cf11c246d356ef354c130a

9. Verdezoto N, Grönvall E. On preventive blood pressure self-monitoring at home. Cogn Technol Work. 2016;18(2):267–85.

10. Rapp A, Cena F. Personal informatics for everyday life: How users without prior self-tracking experience engage with personal data. Int J Hum Comput Stud. 2016;94:1–17.

11. Clinch S, Davies N, Mikusz M, Metzger P, Langheinrich M, Schmidt A, et al. Collecting Shared Experiences through Lifelogging: Lessons Learned. IEEE Pervasive Comput. 2016;15(1):58–67.

12. Hermsen S, Moons J, Kerkhof P, Wiekens C, De Groot M. Determinants for Sustained Use of an Activity Tracker: Observational Study. JMIR MHealth UHealth. 2017 Oct 30;5(10):e164.

13. Faust L, Hachen D, Lizardo O, Chawla NV. Quantifying Subjective Well-Being Using Trends in Weekend Activity. In: 2018 IEEE International Conference on Healthcare Informatics (ICHI) [Internet]. New York, NY: IEEE; 2018 [cited 2025 Aug 2]. p. 123–9. Available from: https://ieeexplore.ieee.org/document/8419354/

14. Chrisinger BW, King AC. Stress experiences in neighborhood and social environments (SENSE): A pilot study to integrate the quantified self with citizen science to improve the built environment and health. Int J Health Geogr [Internet]. 2018;17(1). Available from:

https://www.scopus.com/inward/record.uri?eid=2-s2.0-85048138004&doi=10.1186%2fs12942-018-0140-1&partnerID=40&md5=f98935b05eaeee99a06c6b277ec5667b

15. Kooiman TJM, De Groot M, Hoogenberg K, Krijnen WP, Van Der Schans CP, Kooy A. Self-tracking of Physical Activity in People With Type 2 Diabetes: A Randomized Controlled Trial. CIN Comput Inform Nurs. 2018 July;36(7):340–9.

16. Huppert F, Kranz M, Hoelzl G. Would I Lie to You - Would You Notice? In: Yin Y., Li Y., Gao H., Zhang J., editors. Int Conf Mob Comput, Appl Serv [Internet]. ICST; 2019. p. 230–43. Available from: https://www.scopus.com/inward/record.uri?eid=2-s2.0-85161891696&doi=10.1007%2f978-3-030-28468-8_17&partnerID=40&md5=fc7bfb5fa9d378012b3db1e6ea527498

17. Ensari I, Pichon A, Lipsky-Gorman S, Bakken S, Elhadad N. Augmenting the clinical data sources for enigmatic diseases: A cross-sectional study of self-tracking data and clinical documentation in endometriosis. Appl Clin Inform. 2020;11(5):769–84.

18. Ruckenstein M. Visualized and interacted life: Personal analytics and engagements with data doubles. Societies. 2014;4(1):68–84.

19. Baron KG, Abbott S, Jao N, Manalo N, Mullen R. Orthosomnia: Are Some Patients Taking the Quantified Self Too Far? J Clin Sleep Med. 2017 Feb 15;13(02):351–4.

20. Kristensen DB, Ruckenstein M. Co-evolving with self-tracking technologies. New Media Soc. 2018;20(10):3624–40.

21. Vesnic-Alujevic L, Breitegger M, Guimaraes Pereira Â. "Do-It-Yourself" Healthcare? Quality of Health and Healthcare Through Wearable Sensors. Sci Eng Ethics. 2016;1–18.

22. Chiodo S. Quantified Self as Epistemological Anarchism. Philosophia. 2022 Sept;50(4):1665–85.

23. De Christofaro D, Chiodo S. Quantified Sleep: Self-Tracking Technologies and the Reshaping of 21st-Century Subjectivity. Hist Soc Res. 2023;48:176193.

24. Call SR, Jensen JT, Barbour JB. Quantifying the self with others. Big Data Soc [Internet]. 2024;11(2). Available from: https://www.scopus.com/inward/record.uri?eid=2-s2.0-85191294585&doi=10.1177%2f20539517241247831&partnerID=40&md5=48e6e0b6f58dbb72d4656033fe5d5010

25. Whitson JR. Gaming the quantified self. Surveill Soc. 2013;11(1–2):163–76.

26. Wilmott C, Fraser E, Lammes S. *'I am he. I am he. Siri rules'* : Work and play with the Apple Watch. Eur J Cult Stud. 2018 Feb;21(1):78–95.

27. Hepworth K. A Panopticon on My Wrist: The Biopower of Big Data Visualization for Wearables. Des Cult. 2019;11(3):323–44.

28. Swan M. The Quantified Self: Fundamental Disruption in Big Data Science and Biological Discovery. Big Data [Internet]. 2013 [cited 2015 Mar 15];1(2). Available from: http://quantifiedself.com/about/

29. Choe EK, Lee NB, Lee B, Pratt W, Kientz J a. Understanding quantified-selfers' practices in collecting and exploring personal data. Proc 32nd Annu ACM Conf Hum Factors Comput Syst - CHI 14. 2014;1143–52.

30. Nafus D, Sherman J. This one does not go up to 11: The quantified self movement as an alternative big data practice. Int J Commun. 2014;8(1).

31. Sharon T, Zandbergen D. From data fetishism to quantifying selves: Self-tracking practices and the other values of data. New Media Soc. 2017;19(11):1695–709.

32. Dolejšová M, Kera D. Soylent Diet Self-Experimentation: Design Challenges in Extreme Citizen Science Projects. In: Proceedings of the 2017 ACM Conference on Computer Supported Cooperative Work and Social Computing [Internet]. Portland Oregon USA: ACM; 2017 [cited 2025 Apr 18]. p. 2112–23. Available from: https://dl.acm.org/doi/10.1145/2998181.2998365

33. Heyen NB. From self-tracking to self-expertise: The production of self-related knowledge by doing personal science. Public Underst Sci. 2019;1–15.

34. Robards B, Lyall B, Moran C. Confessional data selfies and intimate digital traces. New Media Soc. 2021;23(9):2616–33.

35. Vermeer L. From Diaries to Data Doubles. Self-Tracking in Dutch Diaries (1780–1940). Life Writ. 2022 Apr 3;19(2):215–40.

36. Zillien N, Wettmann N, Peper F. Sleep Experiments. Knowledge Production through Self-Tracking. Hist Soc Res. 2023;48:157175.

37. Rigal A. On the cult of the individual: The Quantified Self public gatherings, self-tracking, and individualism. Curr Sociol. 2024 Sept 22;00113921241275680.

38. Magnuson V, Wang Y, Schork N. Normalizing sleep quality disturbed by psychiatric polypharmacy: a single patient open trial (SPOT). F1000Research. 2016 Sept 5;5:132.

39. Riggare S, Unruh KT, Sturr J, Domingos J, Stamford JA, Svenningsson P, et al. Patient-driven N-of-1 in Parkinson's disease: Lessons learned from a placebo-controlled study of the effect of nicotine on dyskinesia. Methods Inf Med. 2017;56(MethodsOpen):123–8.

40. Lee V, Thurston T, Thurston C. A Comparison of Discovered Regularities in Blood Glucose Readings across Two Data Collection Approaches Used with a Type 1 Diabetic Youth. Methods Inf Med. 2017;56(99):84–91.

41. Marcengo A, Sabena E, Crea A. Mindfulness and asynchronous neurofeedback: Coping with mind wandering. In: Antona M., Stephanidis C., editors. Lect Notes Comput Sci [Internet]. Springer Verlag; 2017. p. 549–61. Available from: https://www.scopus.com/inward/record.uri?eid=2-s2.0-85025135475&doi=10.1007%2f978-3-319-58700-4_45&partnerID=40&md5=2f9d48ee4c63979319e90df9835ad700

42. Christiansen TB, Kristensen DB, Larsen JE. The 1-person laboratory of the quantified self community. In: Metr Cult: Ontol of Self-Track Pract [Internet]. Emerald Group Publishing Ltd.; 2018. p. 97–115. Available from: https://www.scopus.com/inward/record.uri?eid=2-s2.0-85115775057&doi=10.1108%2f978-1-78743-289-520181006&partnerID=40&md5=91a6ca196f55de08baf78e0abd6352b1

43. Grant AD, Wolf GI. Free-Living Humans Cross Cardiovascular Disease Risk Categories Due to Daily Rhythms in Cholesterol and Triglycerides. J Circadian Rhythms. 2019;17(1):1–13.

44. White G, Liang Z, Clarke S. A Quantified-Self Framework for Exploring and Enhancing Personal Productivity. In: 2019 International Conference on Content-Based Multimedia Indexing (CBMI) [Internet]. Dublin, Ireland: IEEE; 2019 [cited 2025 Aug 2]. p. 1–6. Available from: https://ieeexplore.ieee.org/document/8877475/

45. Tan SHA, Smith TEL. An optimal environment for our optimal selves? An autoethnographic account of self-tracking personal exposure to air pollution. Area. 2021;53(2):353–61.

46. Butte KD, Bahmani A, Butte AJ, Li X, Snyder MP. Five-year pediatric use of a digital wearable fitness device: Lessons from a pilot case study. JAMIA Open [Internet]. 2021;4(3). Available from: https://www.scopus.com/inward/record.uri?eid=2-s2.0-85118938583&doi=10.1093%2fjamiaopen%2fooab054&partnerID=40&md5=fa94591ee3b7f677278f9617a5032d9a
